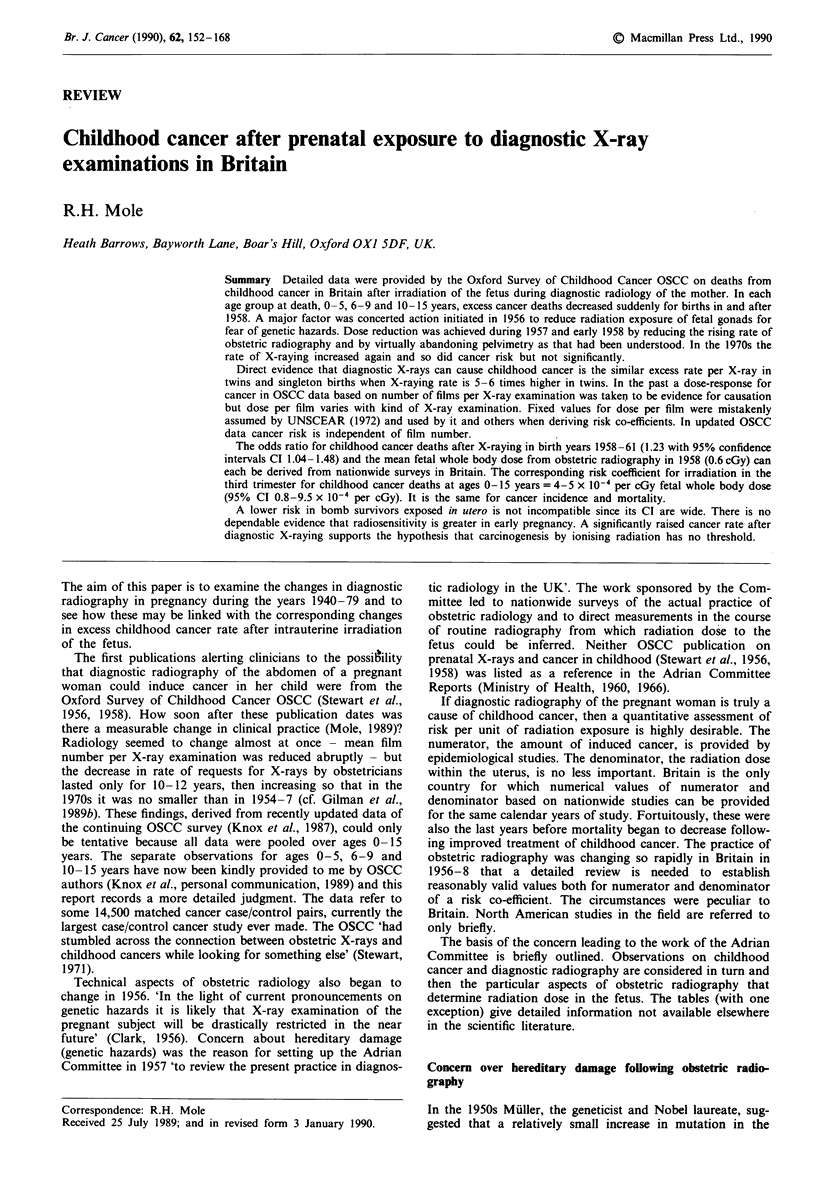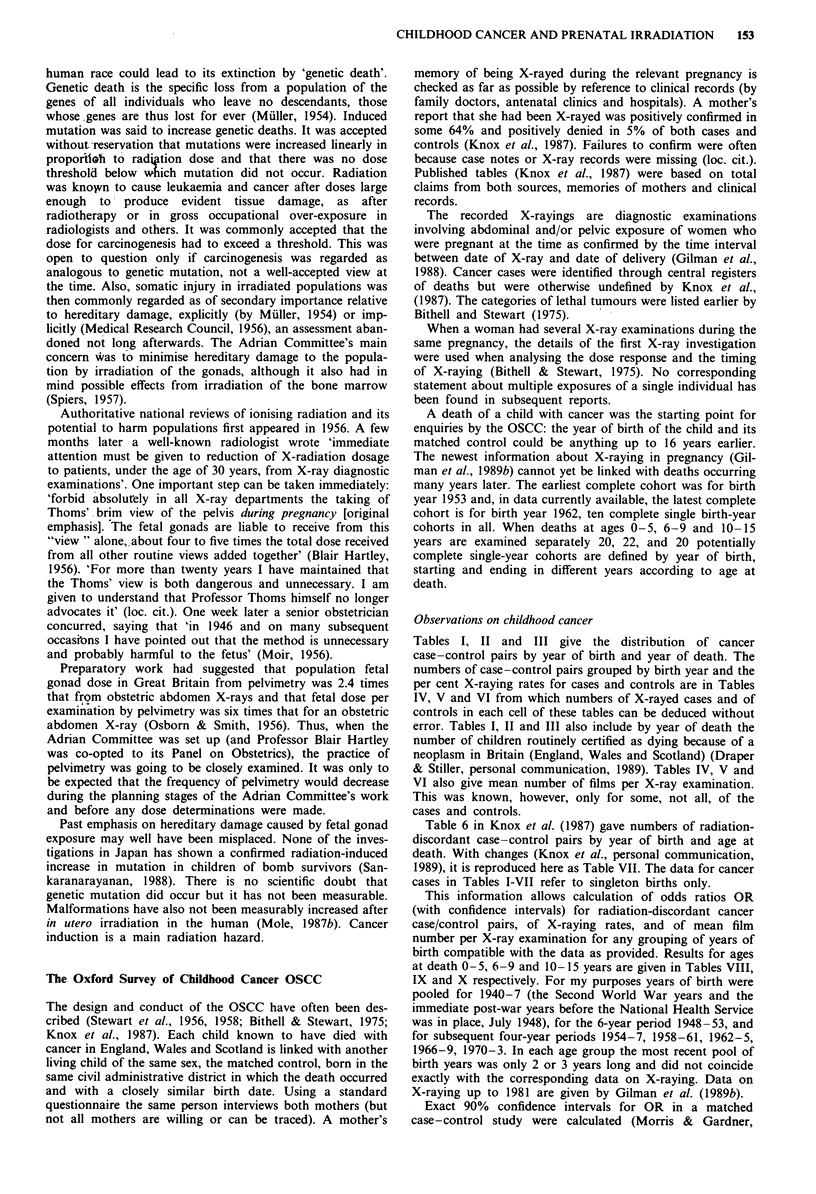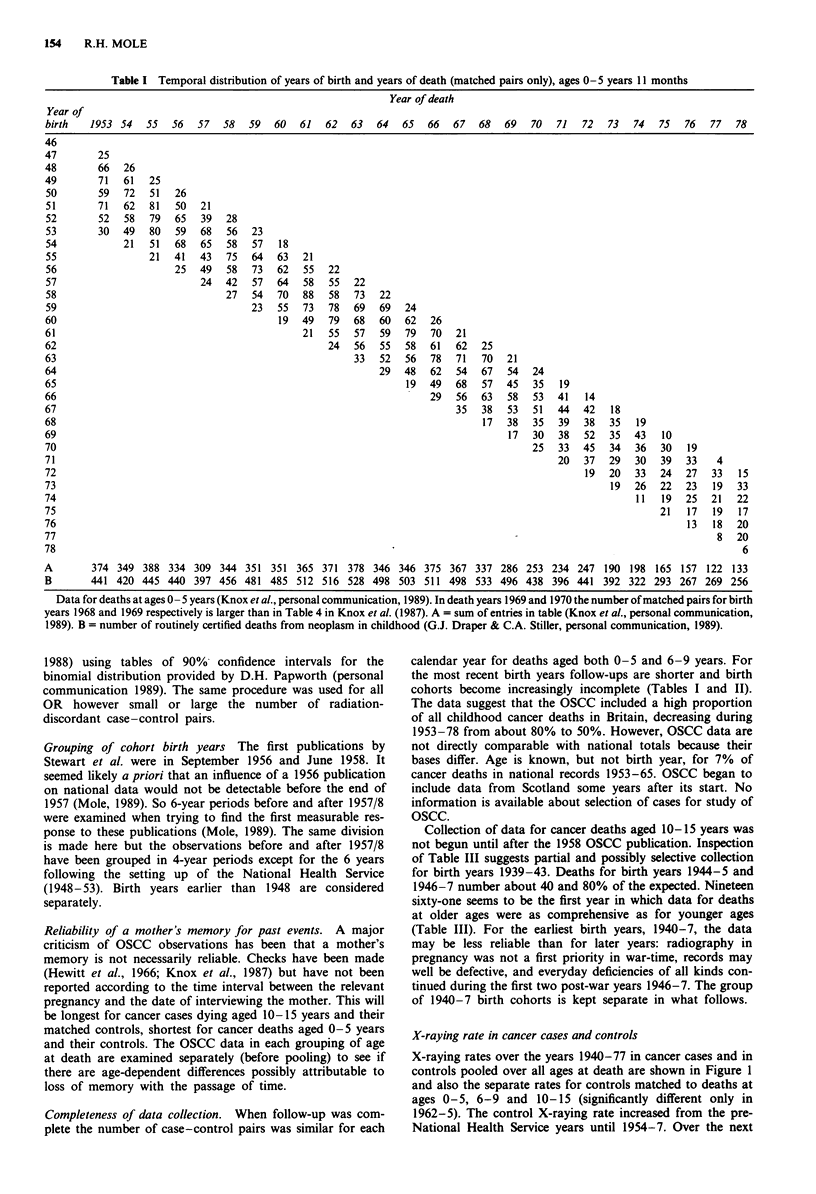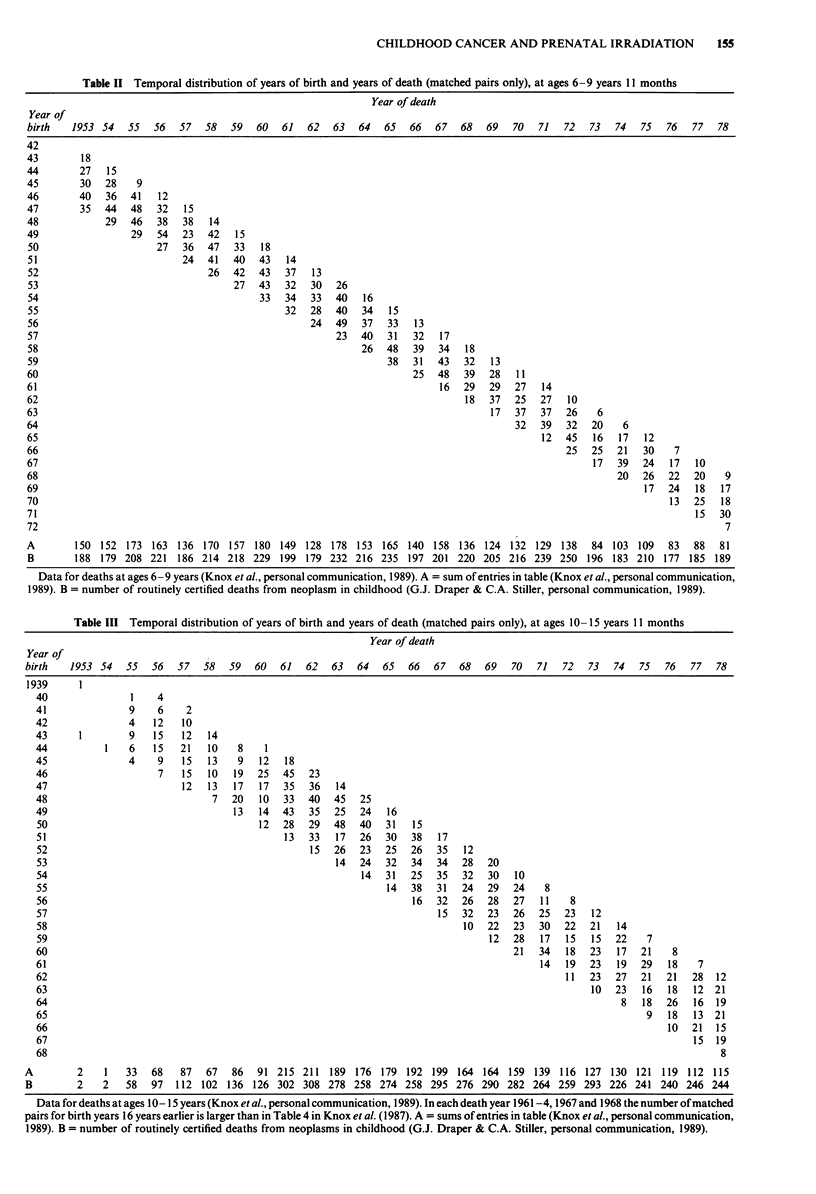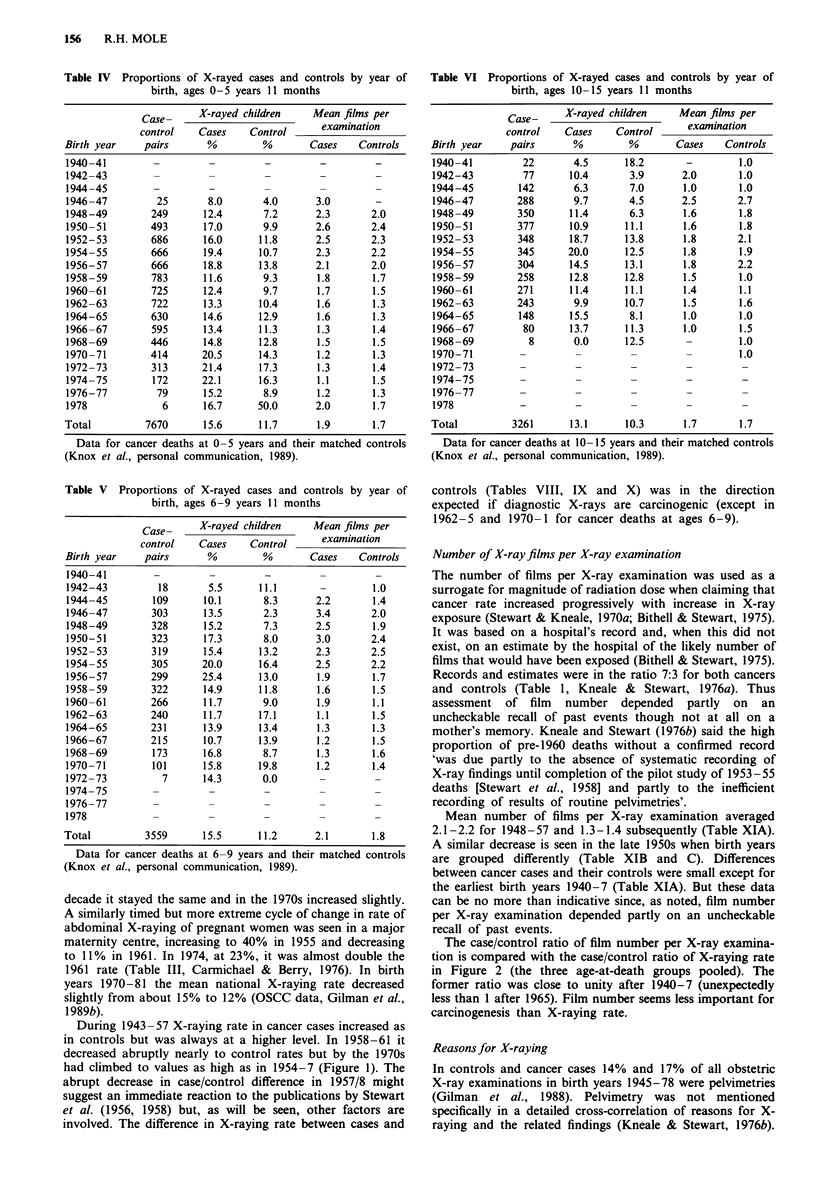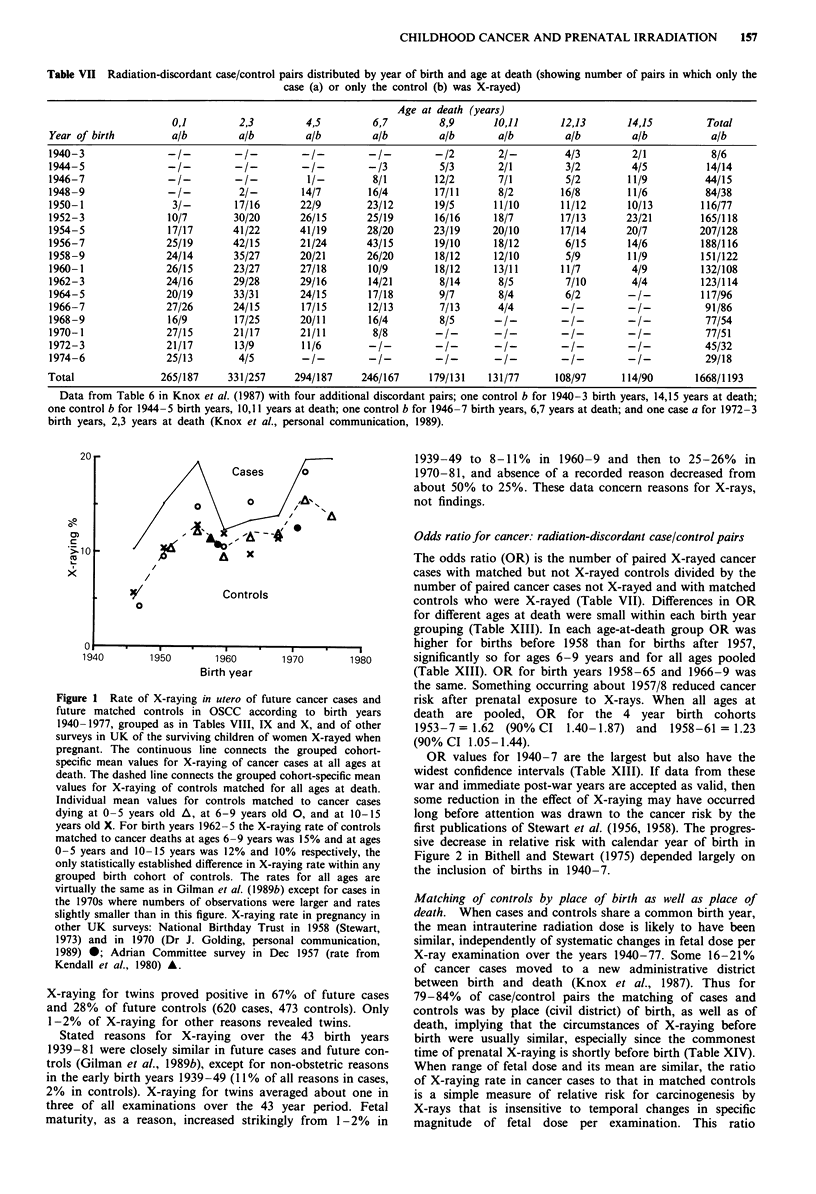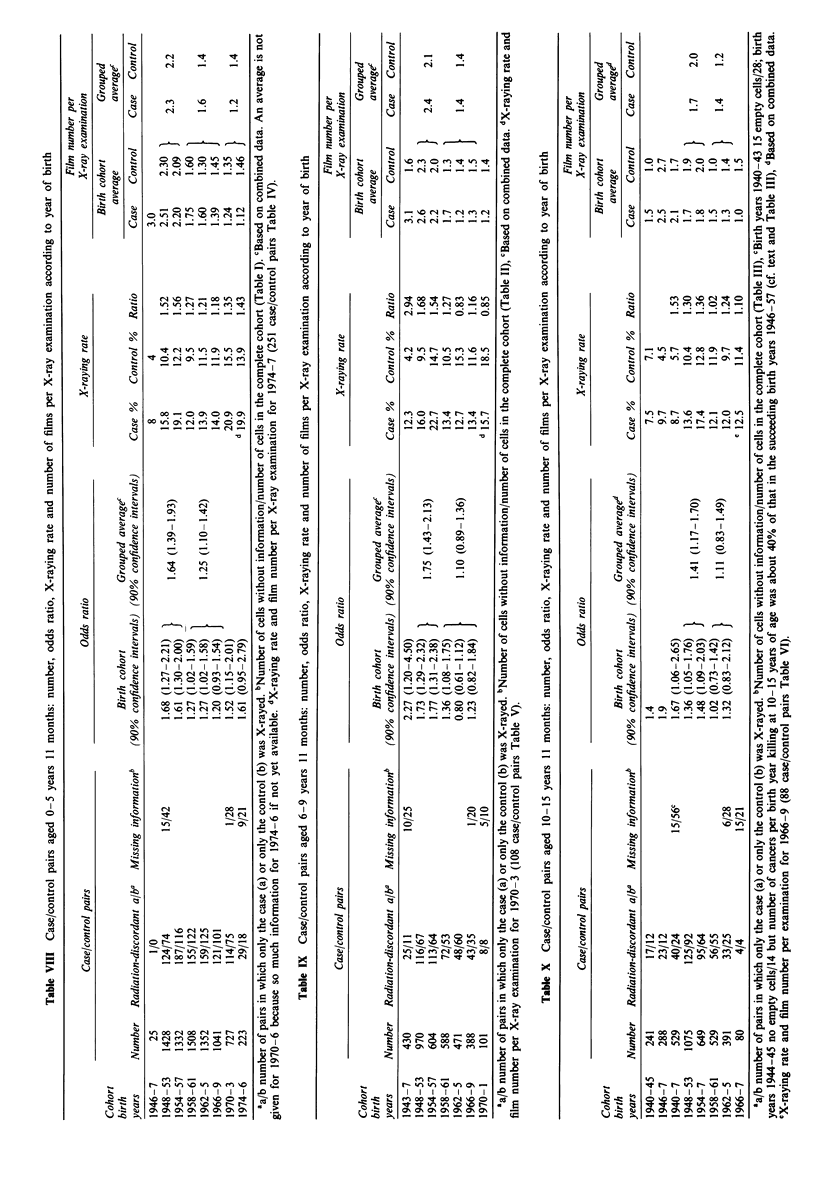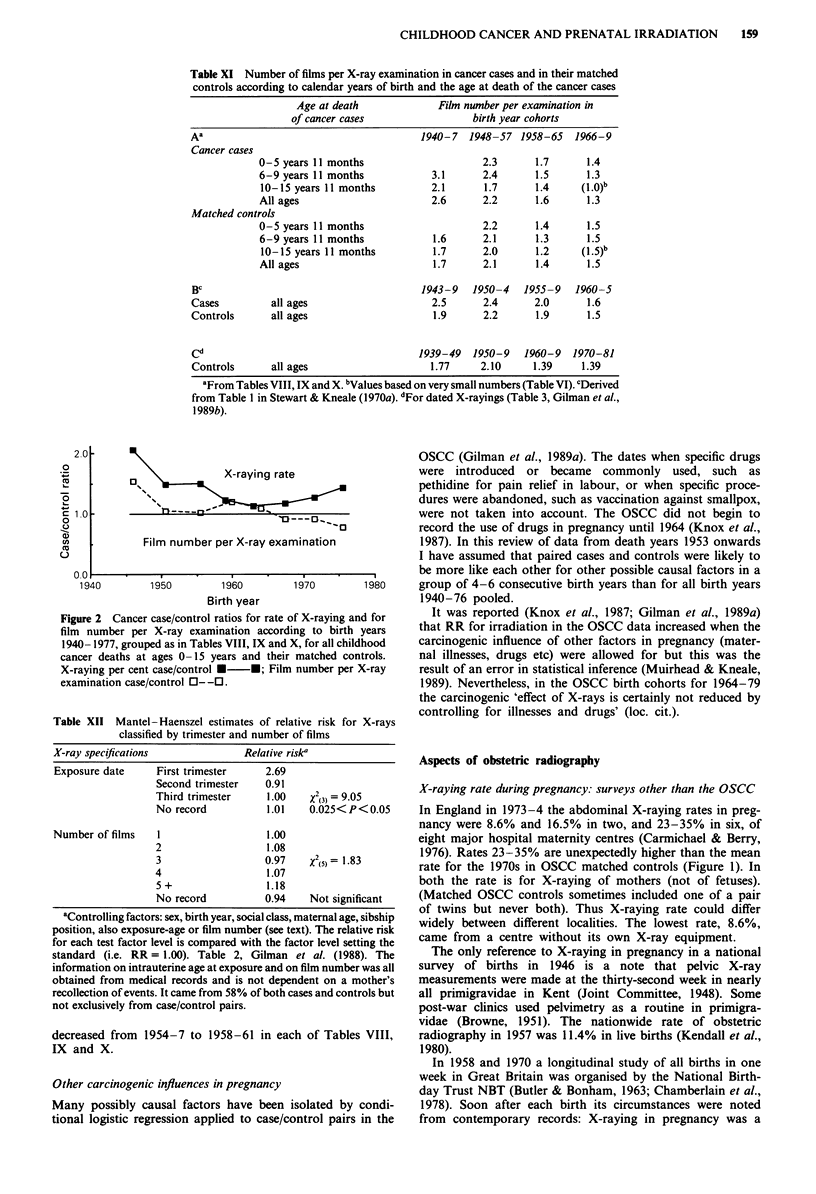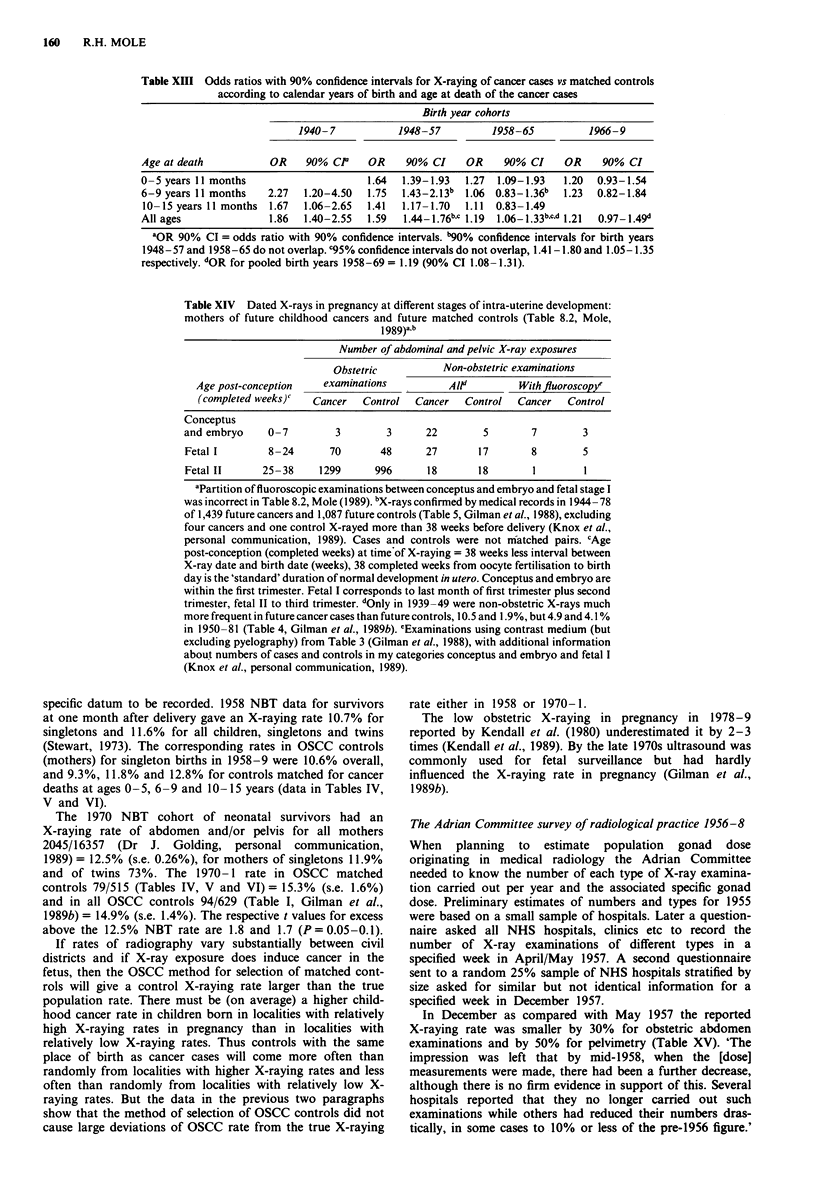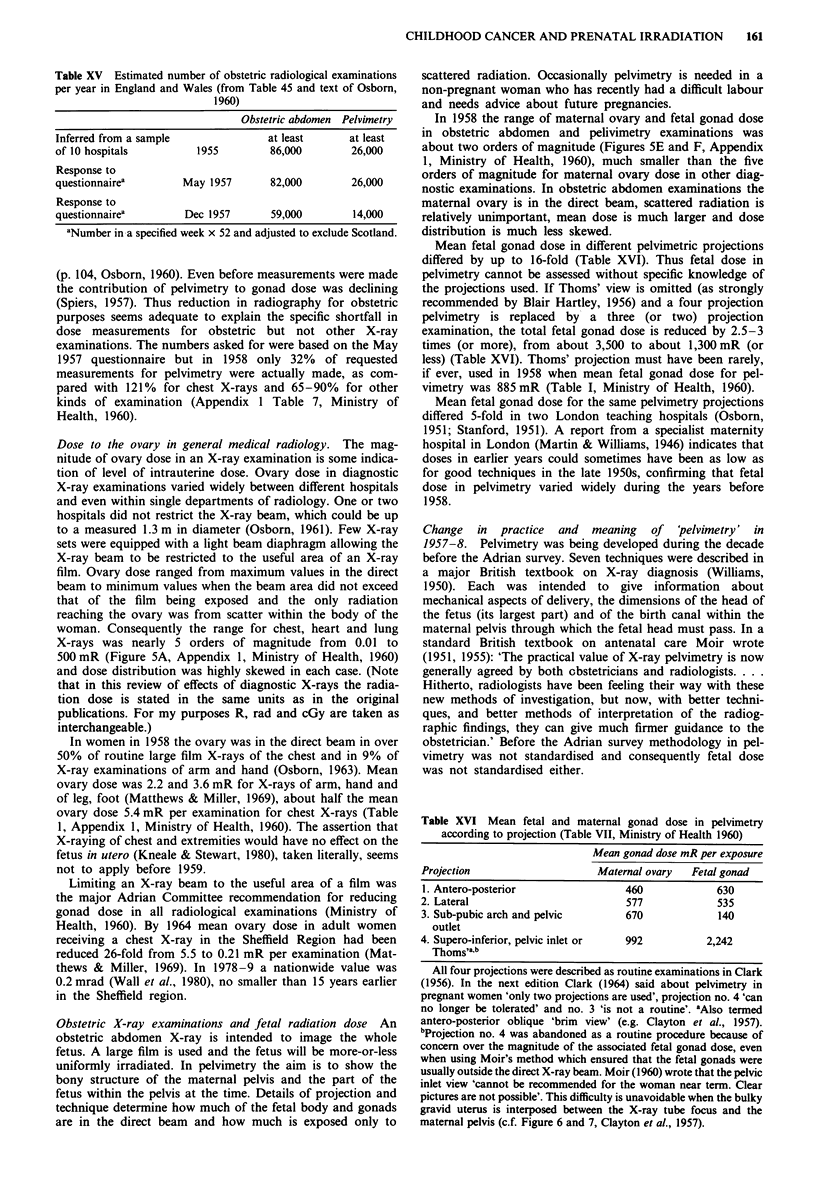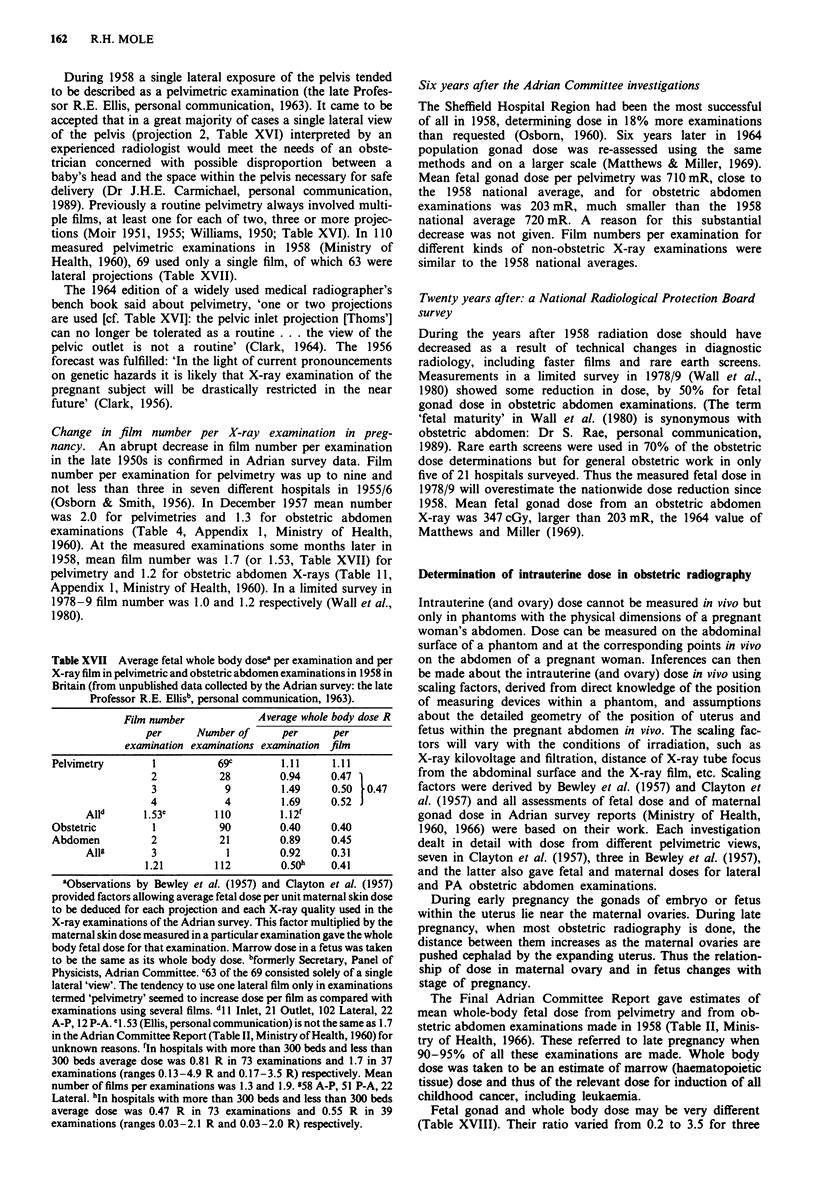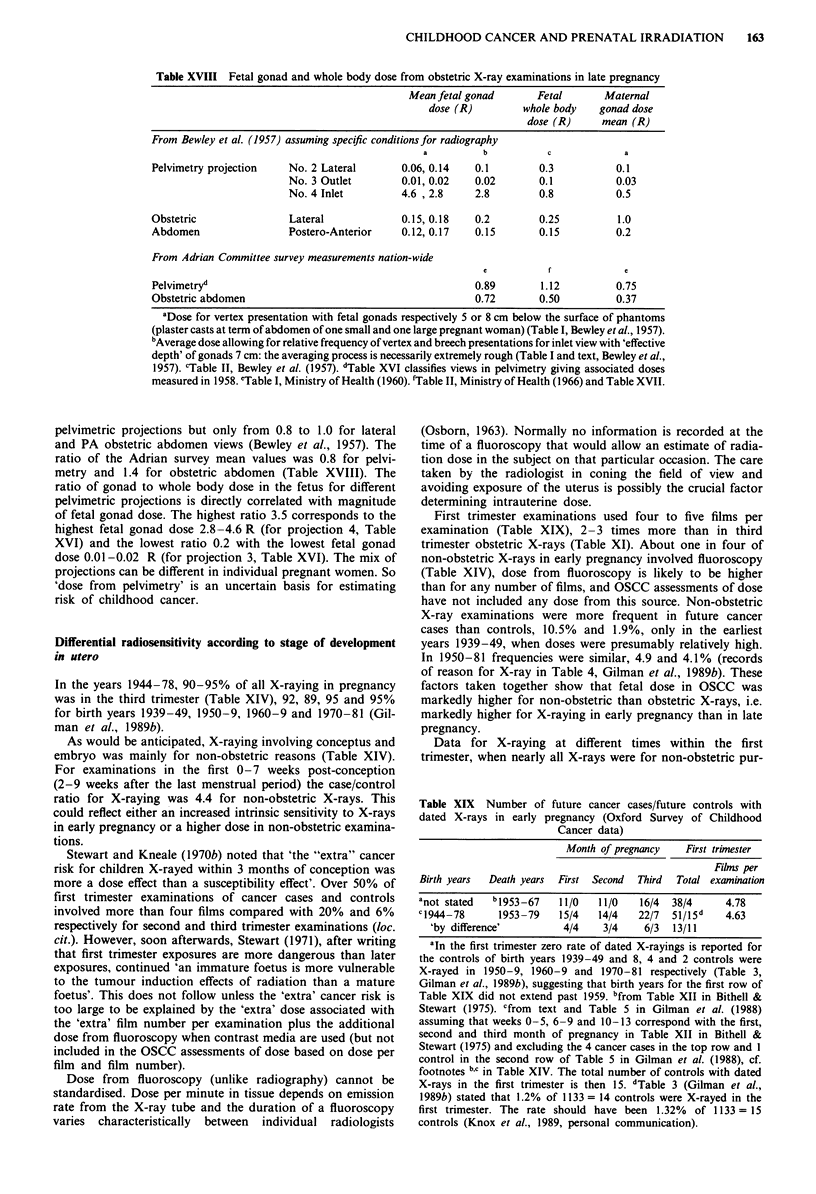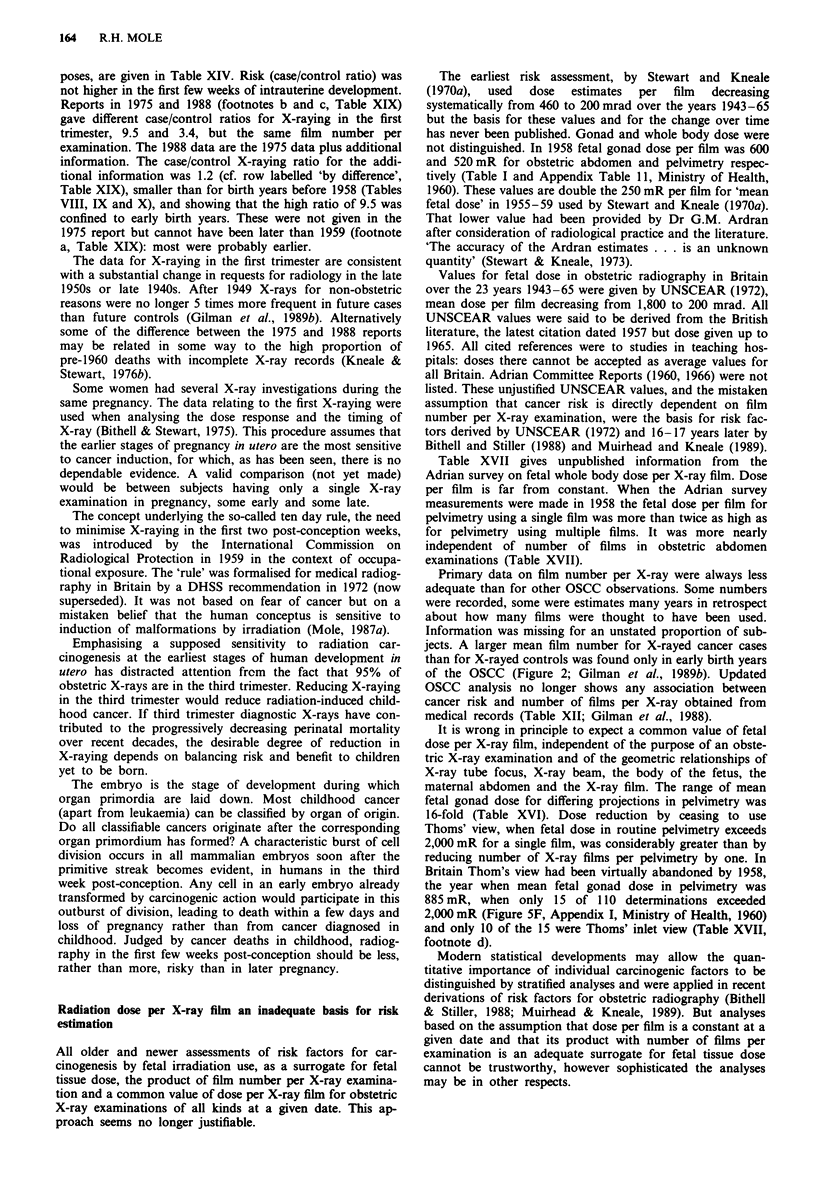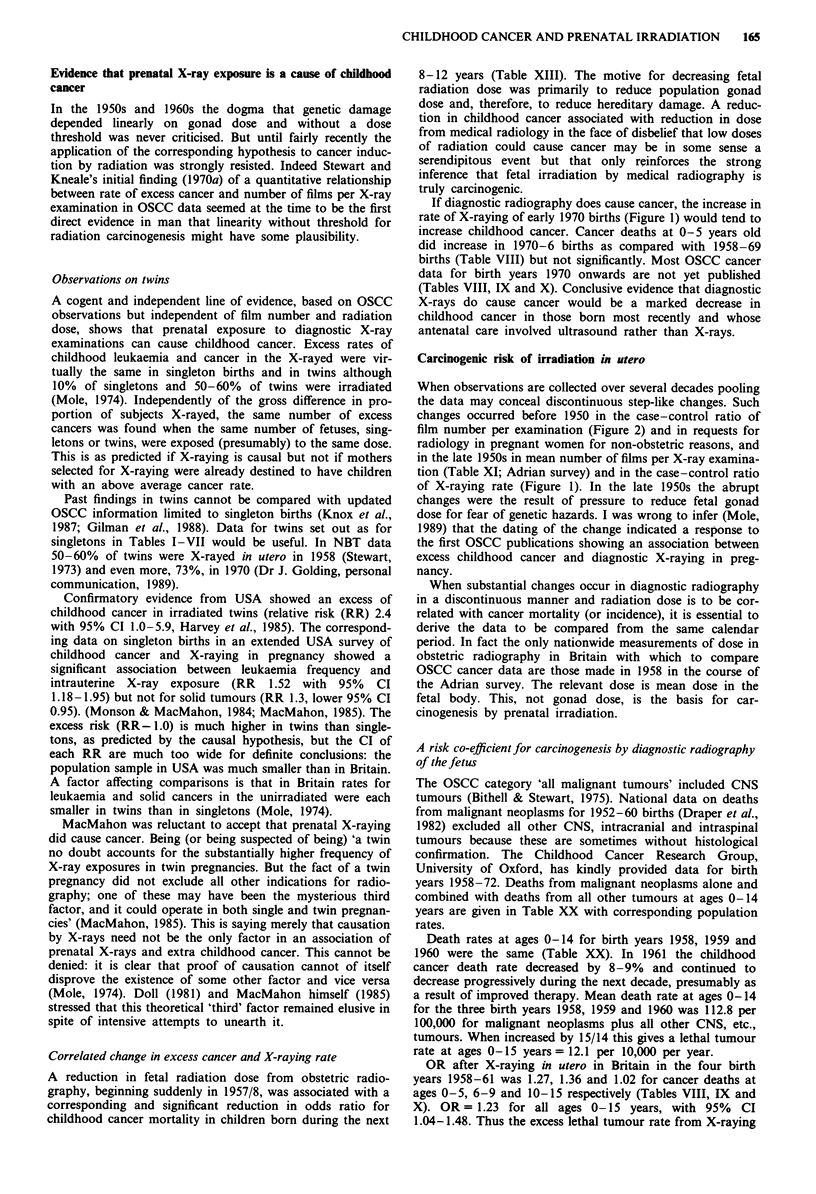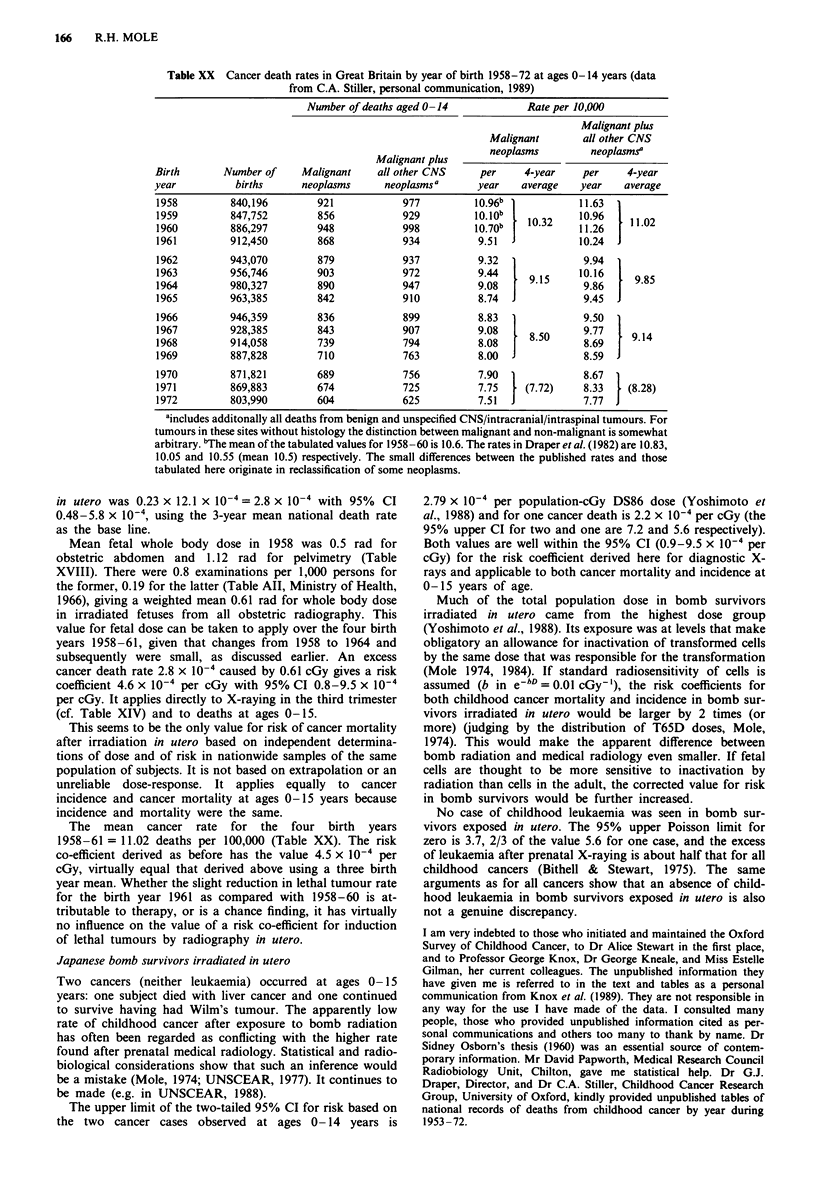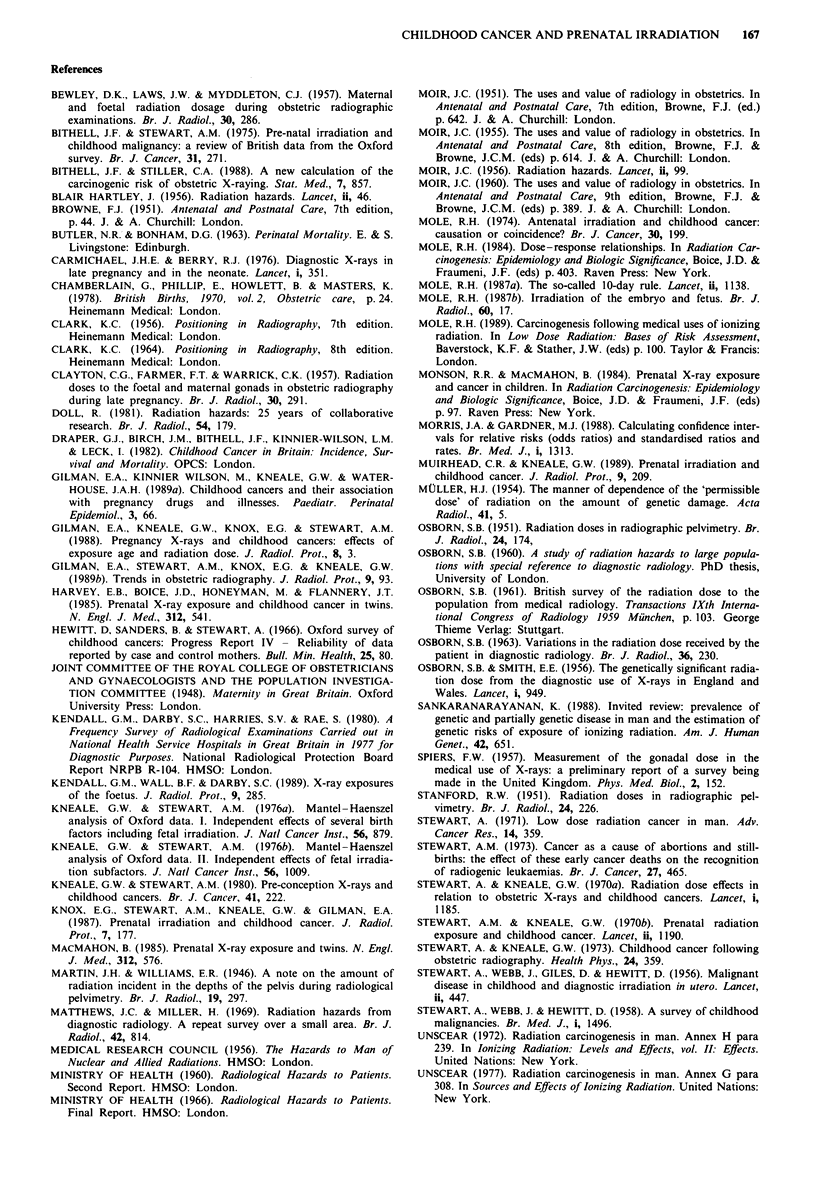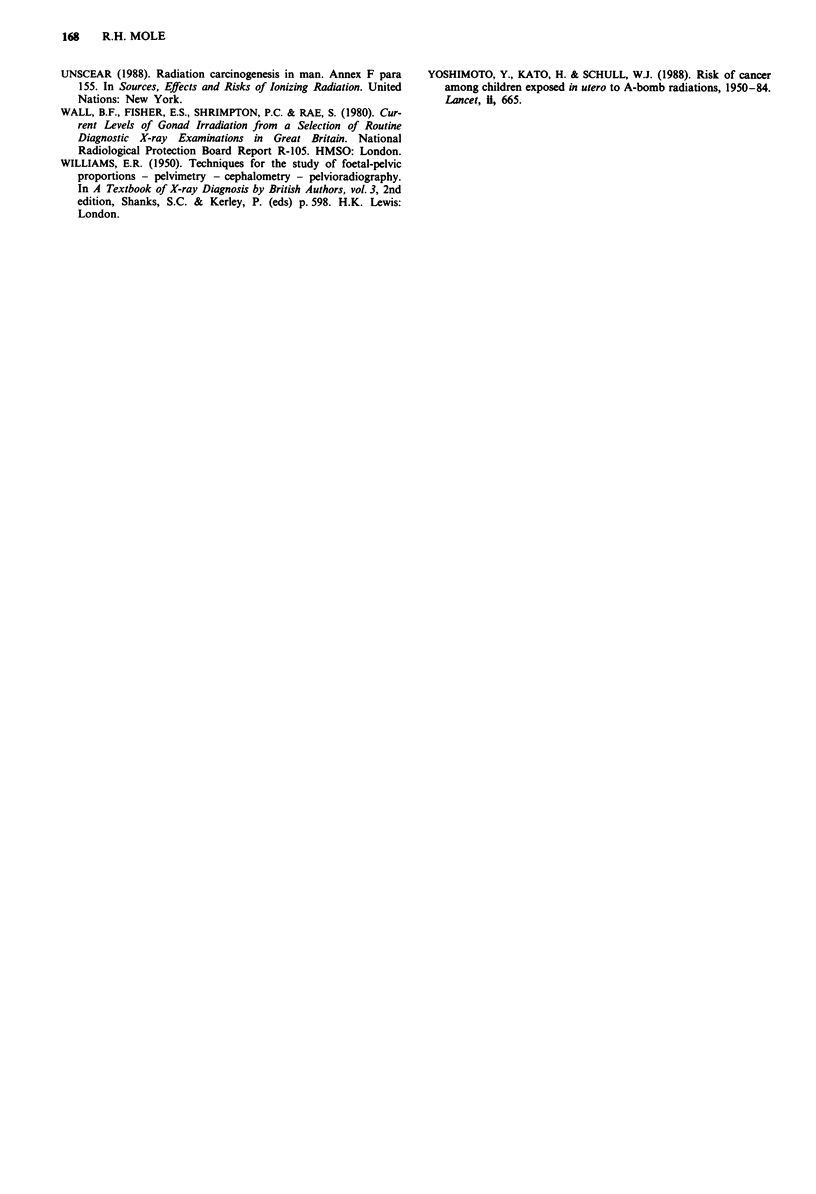# Childhood cancer after prenatal exposure to diagnostic X-ray examinations in Britain.

**DOI:** 10.1038/bjc.1990.249

**Published:** 1990-07

**Authors:** R. H. Mole

**Affiliations:** Heath Barrows, Boar's Hill, Oxford, UK.

## Abstract

Detailed data were provided by the Oxford Survey of Childhood Cancer OSCC on deaths from childhood cancer in Britain after irradiation of the fetus during diagnostic radiology of the mother. In each age group at death, 0-5, 6-9 and 10-15 years, excess cancer deaths decreased suddenly for births in and after 1958. A major factor was concerted action initiated in 1956 to reduce radiation exposure of fetal gonads for fear of genetic hazards. Dose reduction was achieved during 1957 and early 1958 by reducing the rising rate of obstetric radiography and by virtually abandoning pelvimetry as that had been understood. In the 1970s the rate of X-raying increased again and so did cancer risk but not significantly. Direct evidence that diagnostic X-rays can cause childhood cancer is the similar excess rate per X-ray in twins and singleton births when X-raying rate is 5-6 times higher in twins. In the past a dose-response for cancer in OSCC data based on number of films per X-ray examination was taken to be evidence for causation but dose per film varies with kind of X-ray examination. Fixed values for dose per film were mistakenly assumed by UNSCEAR (1972) and used by it and others when deriving risk co-efficients. In updated OSCC data cancer risk is independent of film number. The odds ratio for childhood cancer deaths after X-raying in birth years 1958-61 (1.23 with 95% confidence intervals CI 1.04-1.48) and the mean fetal whole body dose from obstetric radiography in 1958 (0.6 cGy) can each be derived from nationwide surveys in Britain. The corresponding risk coefficient for irradiation in the third trimester for childhood cancer deaths at ages 0-15 years = 4-5 x 10(-4) per cGy fetal whole body dose (95% CI 0.8-9.5 x 10(-4) per cGy). It is the same for cancer incidence and mortality. A lower risk in bomb survivors exposed in utero is not incompatible since its CI are wide. There is no dependable evidence that radiosensitivity is greater in early pregnancy. A significantly raised cancer rate after diagnostic X-raying supports the hypothesis that carcinogenesis by ionising radiation has no threshold.


*Br. J. Cancer* (1990), **62**, 152–168 © Macmillan Press Ltd., 1990

REVIEW

### Childhood cancer after prenatal exposure to diagnostic X-ray examinations in Britain

R.H. Mole

*Heath Barrows, Bayworth Lane, Boar's Hill, Oxford OX1 5DF, UK.*

**Summary** Detailed data were provided by the Oxford Survey of Childhood Cancer OSCC on deaths from childhood cancer in Britain after irradiation of the fetus during diagnostic radiology of the mother. In each age group at death, 0–5, 6–9 and 10–15 years, excess cancer deaths decreased suddenly for births in and after 1958. A major factor was concerted action initiated in 1956 to reduce radiation exposure of fetal gonads for fear of genetic hazards. Dose reduction was achieved during 1957 and early 1958 by reducing the rising rate of obstetric radiography and by virtually abandoning pelvimetry as that had been understood. In the 1970s the rate of X-raying increased again and so did cancer risk but not significantly.

Direct evidence that diagnostic X-rays can cause childhood cancer is the similar excess rate per X-ray in twins and singleton births when X-raying rate is 5–6 times higher in twins. In the past a dose-response for cancer in OSCC data based on number of films per X-ray examination was taken to be evidence for causation but dose per film varies with kind of X-ray examination. Fixed values for dose per film were mistakenly assumed by UNSCEAR (1972) and used by it and others when deriving risk co-efficients. In updated OSCC data cancer risk is independent of film number.

The odds ratio for childhood cancer deaths after X-raying in birth years 1958–61 (1.23 with 95% confidence intervals CI 1.04–1.48) and the mean fetal whole body dose from obstetric radiography in 1958 (0.6 cGy) can each be derived from nationwide surveys in Britain. The corresponding risk coefficient for irradiation in the third trimester for childhood cancer deaths at ages 0–15 years = 4–5 $\times 10^{-4}$ per cGy fetal whole body dose (95% CI 0.8–9.5 $\times 10^{-4}$ per cGy). It is the same for cancer incidence and mortality.

A lower risk in bomb survivors exposed *in utero* is not incompatible since its CI are wide. There is no dependable evidence that radiosensitivity is greater in early pregnancy. A significantly raised cancer rate after diagnostic X-raying supports the hypothesis that carcinogenesis by ionising radiation has no threshold.

The aim of this paper is to examine the changes in diagnostic radiography in pregnancy during the years 1940–79 and to see how these may be linked with the corresponding changes in excess childhood cancer rate after intrauterine irradiation of the fetus.

The first publications alerting clinicians to the possibility that diagnostic radiography of the abdomen of a pregnant woman could induce cancer in her child were from the Oxford Survey of Childhood Cancer OSCC (Stewart *et al.*, 1956, 1958). How soon after these publication dates was there a measurable change in clinical practice (Mole, 1989)? Radiology seemed to change almost at once – mean film number per X-ray examination was reduced abruptly – but the decrease in rate of requests for X-rays by obstetricians lasted only for 10–12 years, then increasing so that in the 1970s it was no smaller than in 1954–7 (cf. Gilman *et al.*, 1989*b*). These findings, derived from recently updated data of the continuing OSCC survey (Knox *et al.*, 1987), could only be tentative because all data were pooled over ages 0–15 years. The separate observations for ages 0–5, 6–9 and 10–15 years have now been kindly provided to me by OSCC authors (Knox *et al.*, personal communication, 1989) and this report records a more detailed judgment. The data refer to some 14,500 matched cancer case/control pairs, currently the largest case/control cancer study ever made. The OSCC 'had stumbled across the connection between obstetric X-rays and childhood cancers while looking for something else' (Stewart, 1971).

Technical aspects of obstetric radiology also began to change in 1956. 'In the light of current pronouncements on genetic hazards it is likely that X-ray examination of the pregnant subject will be drastically restricted in the near future' (Clark, 1956). Concern about hereditary damage (genetic hazards) was the reason for setting up the Adrian Committee in 1957 'to review the present practice in diagnostic radiology in the UK'. The work sponsored by the Committee led to nationwide surveys of the actual practice of obstetric radiology and to direct measurements in the course of routine radiography from which radiation dose to the fetus could be inferred. Neither OSCC publication on prenatal X-rays and cancer in childhood (Stewart *et al.*, 1956, 1958) was listed as a reference in the Adrian Committee Reports (Ministry of Health, 1960, 1966).

If diagnostic radiography of the pregnant woman is truly a cause of childhood cancer, then a quantitative assessment of risk per unit of radiation exposure is highly desirable. The numerator, the amount of induced cancer, is provided by epidemiological studies. The denominator, the radiation dose within the uterus, is no less important. Britain is the only country for which numerical values of numerator and denominator based on nationwide studies can be provided for the same calendar years of study. Fortuitously, these were also the last years before mortality began to decrease following improved treatment of childhood cancer. The practice of obstetric radiography was changing so rapidly in Britain in 1956–8 that a detailed review is needed to establish reasonably valid values both for numerator and denominator of a risk co-efficient. The circumstances were peculiar to Britain. North American studies in the field are referred to only briefly.

The basis of the concern leading to the work of the Adrian Committee is briefly outlined. Observations on childhood cancer and diagnostic radiography are considered in turn and then the particular aspects of obstetric radiography that determine radiation dose in the fetus. The tables (with one exception) give detailed information not available elsewhere in the scientific literature.

### Concern over hereditary damage following obstetric radiography

In the 1950s Müller, the geneticist and Nobel laureate, suggested that a relatively small increase in mutation in the

Correspondence: R.H. Mole
Received 25 July 1989; and in revised form 3 January 1990.

human race could lead to its extinction by 'genetic death'. Genetic death is the specific loss from a population of the genes of all individuals who leave no descendants, those whose genes are thus lost for ever (Müller, 1954). Induced mutation was said to increase genetic deaths. It was accepted without reservation that mutations were increased linearly in proportion to radiation dose and that there was no dose threshold below which mutation did not occur. Radiation was known to cause leukaemia and cancer after doses large enough to produce evident tissue damage, as after radiotherapy or in gross occupational over-exposure in radiologists and others. It was commonly accepted that the dose for carcinogenesis had to exceed a threshold. This was open to question only if carcinogenesis was regarded as analogous to genetic mutation, not a well-accepted view at the time. Also, somatic injury in irradiated populations was then commonly regarded as of secondary importance relative to hereditary damage, explicitly (by Müller, 1954) or implicitly (Medical Research Council, 1956), an assessment abandoned not long afterwards. The Adrian Committee's main concern was to minimise hereditary damage to the population by irradiation of the gonads, although it also had in mind possible effects from irradiation of the bone marrow (Spiers, 1957).

Authoritative national reviews of ionising radiation and its potential to harm populations first appeared in 1956. A few months later a well-known radiologist wrote 'immediate attention must be given to reduction of X-radiation dosage to patients, under the age of 30 years, from X-ray diagnostic examinations'. One important step can be taken immediately: 'forbid absolutely in all X-ray departments the taking of Thoms' brim view of the pelvis *during pregnancy* [original emphasis]. The fetal gonads are liable to receive from this "view " alone, about four to five times the total dose received from all other routine views added together' (Blair Hartley, 1956). 'For more than twenty years I have maintained that the Thoms' view is both dangerous and unnecessary. I am given to understand that Professor Thoms himself no longer advocates it' (loc. cit.). One week later a senior obstetrician concurred, saying that 'in 1946 and on many subsequent occasions I have pointed out that the method is unnecessary and probably harmful to the fetus' (Moir, 1956).

Preparatory work had suggested that population fetal gonad dose in Great Britain from pelvimetry was 2.4 times that from obstetric abdomen X-rays and that fetal dose per examination by pelvimetry was six times that for an obstetric abdomen X-ray (Osborn & Smith, 1956). Thus, when the Adrian Committee was set up (and Professor Blair Hartley was co-opted to its Panel on Obstetrics), the practice of pelvimetry was going to be closely examined. It was only to be expected that the frequency of pelvimetry would decrease during the planning stages of the Adrian Committee's work and before any dose determinations were made.

Past emphasis on hereditary damage caused by fetal gonad exposure may well have been misplaced. None of the investigations in Japan has shown a confirmed radiation-induced increase in mutation in children of bomb survivors (Sankaranarayanan, 1988). There is no scientific doubt that genetic mutation did occur but it has not been measurable. Malformations have also not been measurably increased after *in utero* irradiation in the human (Mole, 1987*b*). Cancer induction is a main radiation hazard.

### The Oxford Survey of Childhood Cancer OSCC

The design and conduct of the OSCC have often been described (Stewart *et al.*, 1956, 1958; Bithell & Stewart, 1975; Knox *et al.*, 1987). Each child known to have died with cancer in England, Wales and Scotland is linked with another living child of the same sex, the matched control, born in the same civil administrative district in which the death occurred and with a closely similar birth date. Using a standard questionnaire the same person interviews both mothers (but not all mothers are willing or can be traced). A mother's memory of being X-rayed during the relevant pregnancy is checked as far as possible by reference to clinical records (by family doctors, antenatal clinics and hospitals). A mother's report that she had been X-rayed was positively confirmed in some 64% and positively denied in 5% of both cases and controls (Knox *et al.*, 1987). Failures to confirm were often because case notes or X-ray records were missing (loc. cit.). Published tables (Knox *et al.*, 1987) were based on total claims from both sources, memories of mothers and clinical records.

The recorded X-rayings are diagnostic examinations involving abdominal and/or pelvic exposure of women who were pregnant at the time as confirmed by the time interval between date of X-ray and date of delivery (Gilman *et al.*, 1988). Cancer cases were identified through central registers of deaths but were otherwise undefined by Knox *et al.*, (1987). The categories of lethal tumours were listed earlier by Bithell and Stewart (1975).

When a woman had several X-ray examinations during the same pregnancy, the details of the first X-ray investigation were used when analysing the dose response and the timing of X-raying (Bithell & Stewart, 1975). No corresponding statement about multiple exposures of a single individual has been found in subsequent reports.

A death of a child with cancer was the starting point for enquiries by the OSCC: the year of birth of the child and its matched control could be anything up to 16 years earlier. The newest information about X-raying in pregnancy (Gilman *et al.*, 1989*b*) cannot yet be linked with deaths occurring many years later. The earliest complete cohort was for birth year 1953 and, in data currently available, the latest complete cohort is for birth year 1962, ten complete single birth-year cohorts in all. When deaths at ages 0–5, 6–9 and 10–15 years are examined separately 20, 22, and 20 potentially complete single-year cohorts are defined by year of birth, starting and ending in different years according to age at death.

### *Observations on childhood cancer*

Tables I, II and III give the distribution of cancer case–control pairs by year of birth and year of death. The numbers of case–control pairs grouped by birth year and the per cent X-raying rates for cases and controls are in Tables IV, V and VI from which numbers of X-rayed cases and of controls in each cell of these tables can be deduced without error. Tables I, II and III also include by year of death the number of children routinely certified as dying because of a neoplasm in Britain (England, Wales and Scotland) (Draper & Stiller, personal communication, 1989). Tables IV, V and VI also give mean number of films per X-ray examination. This was known, however, only for some, not all, of the cases and controls.

Table 6 in Knox *et al.* (1987) gave numbers of radiation-discordant case–control pairs by year of birth and age at death. With changes (Knox *et al.*, personal communication, 1989), it is reproduced here as Table VII. The data for cancer cases in Tables I-VII refer to singleton births only.

This information allows calculation of odds ratios OR (with confidence intervals) for radiation-discordant cancer case/control pairs, of X-raying rates, and of mean film number per X-ray examination for any grouping of years of birth compatible with the data as provided. Results for ages at death 0–5, 6–9 and 10–15 years are given in Tables VIII, IX and X respectively. For my purposes years of birth were pooled for 1940–7 (the Second World War years and the immediate post-war years before the National Health Service was in place, July 1948), for the 6-year period 1948–53, and for subsequent four-year periods 1954–7, 1958–61, 1962–5, 1966–9, 1970–3. In each age group the most recent pool of birth years was only 2 or 3 years long and did not coincide exactly with the corresponding data on X-raying. Data on X-raying up to 1981 are given by Gilman *et al.* (1989*b*).

Exact 90% confidence intervals for OR in a matched case–control study were calculated (Morris & Gardner,

**Table I** Temporal distribution of years of birth and years of death (matched pairs only), ages 0–5 years 11 months

| Year of birth | Year of death 1953 | 54 | 55 | 56 | 57 | 58 | 59 | 60 | 61 | 62 | 63 | 64 | 65 | 66 | 67 | 68 | 69 | 70 | 71 | 72 | 73 | 74 | 75 | 76 | 77 | 78 |
|---|---|---|---|---|---|---|---|---|---|---|---|---|---|---|---|---|---|---|---|---|---|---|---|---|---|---|
| 46 | | | | | | | | | | | | | | | | | | | | | | | | | | |
| 47 | 25 | | | | | | | | | | | | | | | | | | | | | | | | | |
| 48 | 66 | 26 | | | | | | | | | | | | | | | | | | | | | | | | |
| 49 | 71 | 61 | 25 | | | | | | | | | | | | | | | | | | | | | | | |
| 50 | 59 | 72 | 51 | 26 | | | | | | | | | | | | | | | | | | | | | | |
| 51 | 71 | 62 | 81 | 50 | 21 | | | | | | | | | | | | | | | | | | | | | |
| 52 | 52 | 58 | 79 | 65 | 39 | 28 | | | | | | | | | | | | | | | | | | | | |
| 53 | 30 | 49 | 80 | 59 | 68 | 56 | 23 | | | | | | | | | | | | | | | | | | | |
| 54 | | 21 | 51 | 68 | 65 | 58 | 57 | 18 | | | | | | | | | | | | | | | | | | |
| 55 | | | 21 | 41 | 43 | 75 | 64 | 63 | 21 | | | | | | | | | | | | | | | | | |
| 56 | | | | 25 | 49 | 58 | 73 | 62 | 55 | 22 | | | | | | | | | | | | | | | | |
| 57 | | | | | 24 | 42 | 57 | 64 | 58 | 55 | 22 | | | | | | | | | | | | | | | |
| 58 | | | | | | 27 | 54 | 70 | 88 | 58 | 73 | 22 | | | | | | | | | | | | | | |
| 59 | | | | | | | 23 | 55 | 73 | 78 | 69 | 69 | 24 | | | | | | | | | | | | | |
| 60 | | | | | | | | 19 | 49 | 79 | 68 | 60 | 62 | 26 | | | | | | | | | | | | |
| 61 | | | | | | | | | 21 | 55 | 57 | 59 | 79 | 70 | 21 | | | | | | | | | | | |
| 62 | | | | | | | | | | 24 | 56 | 55 | 58 | 61 | 62 | 25 | | | | | | | | | | |
| 63 | | | | | | | | | | | 33 | 52 | 56 | 78 | 71 | 70 | 21 | | | | | | | | | |
| 64 | | | | | | | | | | | | 29 | 48 | 62 | 54 | 67 | 54 | 24 | | | | | | | | |
| 65 | | | | | | | | | | | | | 19 | 49 | 68 | 57 | 45 | 35 | 19 | | | | | | | |
| 66 | | | | | | | | | | | | | | 29 | 56 | 63 | 58 | 53 | 41 | 14 | | | | | | |
| 67 | | | | | | | | | | | | | | | 35 | 38 | 53 | 51 | 44 | 42 | 18 | | | | | |
| 68 | | | | | | | | | | | | | | | | 17 | 38 | 35 | 39 | 38 | 35 | 19 | | | | |
| 69 | | | | | | | | | | | | | | | | | 17 | 30 | 38 | 52 | 35 | 43 | 10 | | | |
| 70 | | | | | | | | | | | | | | | | | | 25 | 33 | 45 | 34 | 36 | 30 | 19 | | |
| 71 | | | | | | | | | | | | | | | | | | | 20 | 37 | 29 | 30 | 39 | 33 | 4 | |
| 72 | | | | | | | | | | | | | | | | | | | | 19 | 20 | 33 | 24 | 27 | 33 | 15 |
| 73 | | | | | | | | | | | | | | | | | | | | | 19 | 26 | 22 | 23 | 19 | 33 |
| 74 | | | | | | | | | | | | | | | | | | | | | | 11 | 19 | 25 | 21 | 22 |
| 75 | | | | | | | | | | | | | | | | | | | | | | | 21 | 17 | 19 | 17 |
| 76 | | | | | | | | | | | | | | | | | | | | | | | | 13 | 18 | 20 |
| 77 | | | | | | | | | | | | | | | | | | | | | | | | | 8 | 20 |
| 78 | | | | | | | | | | | | | | | | | | | | | | | | | | 6 |
| A | 374 | 349 | 388 | 334 | 309 | 344 | 351 | 351 | 365 | 371 | 378 | 346 | 346 | 375 | 367 | 337 | 286 | 253 | 234 | 247 | 190 | 198 | 165 | 157 | 122 | 133 |
| B | 441 | 420 | 445 | 440 | 397 | 456 | 481 | 485 | 512 | 516 | 528 | 498 | 503 | 511 | 498 | 533 | 496 | 438 | 396 | 441 | 392 | 322 | 293 | 267 | 269 | 256 |

Data for deaths at ages 0–5 years (Knox *et al*., personal communication, 1989). In death years 1969 and 1970 the number of matched pairs for birth years 1968 and 1969 respectively is larger than in Table 4 in Knox *et al*. (1987). A = sum of entries in table (Knox *et al*., personal communication, 1989). B = number of routinely certified deaths from neoplasm in childhood (G.J. Draper & C.A. Stiller, personal communication, 1989).

1988) using tables of 90% confidence intervals for the binomial distribution provided by D.H. Papworth (personal communication 1989). The same procedure was used for all OR however small or large the number of radiation-discordant case–control pairs.

*Grouping of cohort birth years* The first publications by Stewart *et al*. were in September 1956 and June 1958. It seemed likely *a priori* that an influence of a 1956 publication on national data would not be detectable before the end of 1957 (Mole, 1989). So 6-year periods before and after 1957/8 were examined when trying to find the first measurable response to these publications (Mole, 1989). The same division is made here but the observations before and after 1957/8 have been grouped in 4-year periods except for the 6 years following the setting up of the National Health Service (1948–53). Birth years earlier than 1948 are considered separately.

*Reliability of a mother's memory for past events.* A major criticism of OSCC observations has been that a mother's memory is not necessarily reliable. Checks have been made (Hewitt *et al*., 1966; Knox *et al*., 1987) but have not been reported according to the time interval between the relevant pregnancy and the date of interviewing the mother. This will be longest for cancer cases dying aged 10–15 years and their matched controls, shortest for cancer deaths aged 0–5 years and their controls. The OSCC data in each grouping of age at death are examined separately (before pooling) to see if there are age-dependent differences possibly attributable to loss of memory with the passage of time.

*Completeness of data collection.* When follow-up was complete the number of case–control pairs was similar for each calendar year for deaths aged both 0–5 and 6–9 years. For the most recent birth years follow-ups are shorter and birth cohorts become increasingly incomplete (Tables I and II). The data suggest that the OSCC included a high proportion of all childhood cancer deaths in Britain, decreasing during 1953–78 from about 80% to 50%. However, OSCC data are not directly comparable with national totals because their bases differ. Age is known, but not birth year, for 7% of cancer deaths in national records 1953–65. OSCC began to include data from Scotland some years after its start. No information is available about selection of cases for study of OSCC.

Collection of data for cancer deaths aged 10–15 years was not begun until after the 1958 OSCC publication. Inspection of Table III suggests partial and possibly selective collection for birth years 1939–43. Deaths for birth years 1944–5 and 1946–7 number about 40 and 80% of the expected. Nineteen sixty-one seems to be the first year in which data for deaths at older ages were as comprehensive as for younger ages (Table III). For the earliest birth years, 1940–7, the data may be less reliable than for later years: radiography in pregnancy was not a first priority in war-time, records may well be defective, and everyday deficiencies of all kinds continued during the first two post-war years 1946–7. The group of 1940–7 birth cohorts is kept separate in what follows.

### *X-raying rate in cancer cases and controls*

X-raying rates over the years 1940–77 in cancer cases and in controls pooled over all ages at death are shown in Figure 1 and also the separate rates for controls matched to deaths at ages 0–5, 6–9 and 10–15 (significantly different only in 1962–5). The control X-raying rate increased from the pre-National Health Service years until 1954–7. Over the next

**Table II** Temporal distribution of years of birth and years of death (matched pairs only), at ages 6–9 years 11 months

| | *Year of death* | | | | | | | | | | | | | | | | | | | | | | | | | |
|---|---|---|---|---|---|---|---|---|---|---|---|---|---|---|---|---|---|---|---|---|---|---|---|---|---|---|
| *Year of birth* | *1953* | *54* | *55* | *56* | *57* | *58* | *59* | *60* | *61* | *62* | *63* | *64* | *65* | *66* | *67* | *68* | *69* | *70* | *71* | *72* | *73* | *74* | *75* | *76* | *77* | *78* |
| 42 | | | | | | | | | | | | | | | | | | | | | | | | | | |
| 43 | 18 | | | | | | | | | | | | | | | | | | | | | | | | | |
| 44 | 27 | 15 | | | | | | | | | | | | | | | | | | | | | | | | |
| 45 | 30 | 28 | 9 | | | | | | | | | | | | | | | | | | | | | | | |
| 46 | 40 | 36 | 41 | 12 | | | | | | | | | | | | | | | | | | | | | | |
| 47 | 35 | 44 | 48 | 32 | 15 | | | | | | | | | | | | | | | | | | | | | |
| 48 | | 29 | 46 | 38 | 38 | 14 | | | | | | | | | | | | | | | | | | | | |
| 49 | | | 29 | 54 | 23 | 42 | 15 | | | | | | | | | | | | | | | | | | | |
| 50 | | | | 27 | 36 | 47 | 33 | 18 | | | | | | | | | | | | | | | | | | |
| 51 | | | | | 24 | 41 | 40 | 43 | 14 | | | | | | | | | | | | | | | | | |
| 52 | | | | | | 26 | 42 | 43 | 37 | 13 | | | | | | | | | | | | | | | | |
| 53 | | | | | | | 27 | 43 | 32 | 30 | 26 | | | | | | | | | | | | | | | |
| 54 | | | | | | | | 33 | 34 | 33 | 40 | 16 | | | | | | | | | | | | | | |
| 55 | | | | | | | | | 32 | 28 | 40 | 34 | 15 | | | | | | | | | | | | | |
| 56 | | | | | | | | | | 24 | 49 | 37 | 33 | 13 | | | | | | | | | | | | |
| 57 | | | | | | | | | | | 23 | 40 | 31 | 32 | 17 | | | | | | | | | | | |
| 58 | | | | | | | | | | | | 26 | 48 | 39 | 34 | 18 | | | | | | | | | | |
| 59 | | | | | | | | | | | | | 38 | 31 | 43 | 32 | 13 | | | | | | | | | |
| 60 | | | | | | | | | | | | | | 25 | 48 | 39 | 28 | 11 | | | | | | | | |
| 61 | | | | | | | | | | | | | | | 16 | 29 | 29 | 27 | 14 | | | | | | | |
| 62 | | | | | | | | | | | | | | | | 18 | 37 | 25 | 27 | 10 | | | | | | |
| 63 | | | | | | | | | | | | | | | | | 17 | 37 | 37 | 26 | 6 | | | | | |
| 64 | | | | | | | | | | | | | | | | | | 32 | 39 | 32 | 20 | 6 | | | | |
| 65 | | | | | | | | | | | | | | | | | | | 12 | 45 | 16 | 17 | 12 | | | |
| 66 | | | | | | | | | | | | | | | | | | | | 25 | 25 | 21 | 30 | 7 | | |
| 67 | | | | | | | | | | | | | | | | | | | | | 17 | 39 | 24 | 17 | 10 | |
| 68 | | | | | | | | | | | | | | | | | | | | | | 20 | 26 | 22 | 20 | 9 |
| 69 | | | | | | | | | | | | | | | | | | | | | | | 17 | 24 | 18 | 17 |
| 70 | | | | | | | | | | | | | | | | | | | | | | | | 13 | 25 | 18 |
| 71 | | | | | | | | | | | | | | | | | | | | | | | | | 15 | 30 |
| 72 | | | | | | | | | | | | | | | | | | | | | | | | | | 7 |
| A | 150 | 152 | 173 | 163 | 136 | 170 | 157 | 180 | 149 | 128 | 178 | 153 | 165 | 140 | 158 | 136 | 124 | 132 | 129 | 138 | 84 | 103 | 109 | 83 | 88 | 81 |
| B | 188 | 179 | 208 | 221 | 186 | 214 | 218 | 229 | 199 | 179 | 232 | 216 | 235 | 197 | 201 | 220 | 205 | 216 | 239 | 250 | 196 | 183 | 210 | 177 | 185 | 189 |

Data for deaths at ages 6–9 years (Knox *et al.*, personal communication, 1989). A = sum of entries in table (Knox *et al.*, personal communication, 1989). B = number of routinely certified deaths from neoplasm in childhood (G.J. Draper & C.A. Stiller, personal communication, 1989).

**Table III** Temporal distribution of years of birth and years of death (matched pairs only), at ages 10–15 years 11 months

| | *Year of death* | | | | | | | | | | | | | | | | | | | | | | | | | |
|---|---|---|---|---|---|---|---|---|---|---|---|---|---|---|---|---|---|---|---|---|---|---|---|---|---|---|
| *Year of birth* | *1953* | *54* | *55* | *56* | *57* | *58* | *59* | *60* | *61* | *62* | *63* | *64* | *65* | *66* | *67* | *68* | *69* | *70* | *71* | *72* | *73* | *74* | *75* | *76* | *77* | *78* |
| 1939 | 1 | | | | | | | | | | | | | | | | | | | | | | | | | |
| 40 | | | 1 | 4 | | | | | | | | | | | | | | | | | | | | | | |
| 41 | | | 9 | 6 | 2 | | | | | | | | | | | | | | | | | | | | | |
| 42 | | | 4 | 12 | 10 | | | | | | | | | | | | | | | | | | | | | |
| 43 | 1 | | 9 | 15 | 12 | 14 | | | | | | | | | | | | | | | | | | | | |
| 44 | | 1 | 6 | 15 | 21 | 10 | 8 | 1 | | | | | | | | | | | | | | | | | | |
| 45 | | | 4 | 9 | 15 | 13 | 9 | 12 | 18 | | | | | | | | | | | | | | | | | |
| 46 | | | | 7 | 15 | 10 | 19 | 25 | 45 | 23 | | | | | | | | | | | | | | | | |
| 47 | | | | | 12 | 13 | 17 | 17 | 35 | 36 | 14 | | | | | | | | | | | | | | | |
| 48 | | | | | | 7 | 20 | 10 | 33 | 40 | 45 | 25 | | | | | | | | | | | | | | |
| 49 | | | | | | | 13 | 14 | 43 | 35 | 25 | 24 | 16 | | | | | | | | | | | | | |
| 50 | | | | | | | | 12 | 28 | 29 | 48 | 40 | 31 | 15 | | | | | | | | | | | | |
| 51 | | | | | | | | | 13 | 33 | 17 | 26 | 30 | 38 | 17 | | | | | | | | | | | |
| 52 | | | | | | | | | | 15 | 26 | 23 | 25 | 26 | 35 | 12 | | | | | | | | | | |
| 53 | | | | | | | | | | | 14 | 24 | 32 | 34 | 34 | 28 | 20 | | | | | | | | | |
| 54 | | | | | | | | | | | | 14 | 31 | 25 | 35 | 32 | 30 | 10 | | | | | | | | |
| 55 | | | | | | | | | | | | | 14 | 38 | 31 | 24 | 29 | 24 | 8 | | | | | | | |
| 56 | | | | | | | | | | | | | | 16 | 32 | 26 | 28 | 27 | 11 | 8 | | | | | | |
| 57 | | | | | | | | | | | | | | | 15 | 32 | 23 | 26 | 25 | 23 | 12 | | | | | |
| 58 | | | | | | | | | | | | | | | | 10 | 22 | 23 | 30 | 22 | 21 | 14 | | | | |
| 59 | | | | | | | | | | | | | | | | | 12 | 28 | 17 | 15 | 15 | 22 | 7 | | | |
| 60 | | | | | | | | | | | | | | | | | | 21 | 34 | 18 | 23 | 17 | 21 | 8 | | |
| 61 | | | | | | | | | | | | | | | | | | | 14 | 19 | 23 | 19 | 29 | 18 | 7 | |
| 62 | | | | | | | | | | | | | | | | | | | | 11 | 23 | 27 | 21 | 21 | 28 | 12 |
| 63 | | | | | | | | | | | | | | | | | | | | | 10 | 23 | 16 | 18 | 12 | 21 |
| 64 | | | | | | | | | | | | | | | | | | | | | | 8 | 18 | 26 | 16 | 19 |
| 65 | | | | | | | | | | | | | | | | | | | | | | | 9 | 18 | 13 | 21 |
| 66 | | | | | | | | | | | | | | | | | | | | | | | | 10 | 21 | 15 |
| 67 | | | | | | | | | | | | | | | | | | | | | | | | | 15 | 19 |
| 68 | | | | | | | | | | | | | | | | | | | | | | | | | | 8 |
| A | 2 | 1 | 33 | 68 | 87 | 67 | 86 | 91 | 215 | 211 | 189 | 176 | 179 | 192 | 199 | 164 | 164 | 159 | 139 | 116 | 127 | 130 | 121 | 119 | 112 | 115 |
| B | 2 | 2 | 58 | 97 | 112 | 102 | 136 | 126 | 302 | 308 | 278 | 258 | 274 | 258 | 295 | 276 | 290 | 282 | 264 | 259 | 293 | 226 | 241 | 240 | 246 | 244 |

Data for deaths at ages 10–15 years (Knox *et al.*, personal communication, 1989). In each death year 1961–4, 1967 and 1968 the number of matched pairs for birth years 16 years earlier is larger than in Table 4 in Knox *et al.* (1987). A = sums of entries in table (Knox *et al.*, personal communication, 1989). B = number of routinely certified deaths from neoplasms in childhood (G.J. Draper & C.A. Stiller, personal communication, 1989).

**Table IV** Proportions of X-rayed cases and controls by year of birth, ages 0–5 years 11 months

| Birth year | Case–control pairs | X-rayed children Cases % | X-rayed children Control % | Mean films per examination Cases | Mean films per examination Controls |
|---|---|---|---|---|---|
| 1940–41 | – | – | – | – | – |
| 1942–43 | – | – | – | – | – |
| 1944–45 | – | – | – | – | – |
| 1946–47 | 25 | 8.0 | 4.0 | 3.0 | – |
| 1948–49 | 249 | 12.4 | 7.2 | 2.3 | 2.0 |
| 1950–51 | 493 | 17.0 | 9.9 | 2.6 | 2.4 |
| 1952–53 | 686 | 16.0 | 11.8 | 2.5 | 2.3 |
| 1954–55 | 666 | 19.4 | 10.7 | 2.3 | 2.2 |
| 1956–57 | 666 | 18.8 | 13.8 | 2.1 | 2.0 |
| 1958–59 | 783 | 11.6 | 9.3 | 1.8 | 1.7 |
| 1960–61 | 725 | 12.4 | 9.7 | 1.7 | 1.5 |
| 1962–63 | 722 | 13.3 | 10.4 | 1.6 | 1.3 |
| 1964–65 | 630 | 14.6 | 12.9 | 1.6 | 1.3 |
| 1966–67 | 595 | 13.4 | 11.3 | 1.3 | 1.4 |
| 1968–69 | 446 | 14.8 | 12.8 | 1.5 | 1.5 |
| 1970–71 | 414 | 20.5 | 14.3 | 1.2 | 1.3 |
| 1972–73 | 313 | 21.4 | 17.3 | 1.3 | 1.4 |
| 1974–75 | 172 | 22.1 | 16.3 | 1.1 | 1.5 |
| 1976–77 | 79 | 15.2 | 8.9 | 1.2 | 1.3 |
| 1978 | 6 | 16.7 | 50.0 | 2.0 | 1.7 |
| Total | 7670 | 15.6 | 11.7 | 1.9 | 1.7 |

Data for cancer deaths at 0–5 years and their matched controls (Knox *et al.*, personal communication, 1989).

**Table V** Proportions of X-rayed cases and controls by year of birth, ages 6–9 years 11 months

| Birth year | Case–control pairs | X-rayed children Cases % | X-rayed children Control % | Mean films per examination Cases | Mean films per examination Controls |
|---|---|---|---|---|---|
| 1940–41 | – | – | – | – | – |
| 1942–43 | 18 | 5.5 | 11.1 | – | 1.0 |
| 1944–45 | 109 | 10.1 | 8.3 | 2.2 | 1.4 |
| 1946–47 | 303 | 13.5 | 2.3 | 3.4 | 2.0 |
| 1948–49 | 328 | 15.2 | 7.3 | 2.5 | 1.9 |
| 1950–51 | 323 | 17.3 | 8.0 | 3.0 | 2.4 |
| 1952–53 | 319 | 15.4 | 13.2 | 2.3 | 2.5 |
| 1954–55 | 305 | 20.0 | 16.4 | 2.5 | 2.2 |
| 1956–57 | 299 | 25.4 | 13.0 | 1.9 | 1.7 |
| 1958–59 | 322 | 14.9 | 11.8 | 1.6 | 1.5 |
| 1960–61 | 266 | 11.7 | 9.0 | 1.9 | 1.1 |
| 1962–63 | 240 | 11.7 | 17.1 | 1.1 | 1.5 |
| 1964–65 | 231 | 13.9 | 13.4 | 1.3 | 1.3 |
| 1966–67 | 215 | 10.7 | 13.9 | 1.2 | 1.5 |
| 1968–69 | 173 | 16.8 | 8.7 | 1.3 | 1.6 |
| 1970–71 | 101 | 15.8 | 19.8 | 1.2 | 1.4 |
| 1972–73 | 7 | 14.3 | 0.0 | – | – |
| 1974–75 | – | – | – | – | – |
| 1976–77 | – | – | – | – | – |
| 1978 | – | – | – | – | – |
| Total | 3559 | 15.5 | 11.2 | 2.1 | 1.8 |

Data for cancer deaths at 6–9 years and their matched controls (Knox *et al.*, personal communication, 1989).

decade it stayed the same and in the 1970s increased slightly. A similarly timed but more extreme cycle of change in rate of abdominal X-raying of pregnant women was seen in a major maternity centre, increasing to 40% in 1955 and decreasing to 11% in 1961. In 1974, at 23%, it was almost double the 1961 rate (Table III, Carmichael & Berry, 1976). In birth years 1970–81 the mean national X-raying rate decreased slightly from about 15% to 12% (OSCC data, Gilman *et al.*, 1989*b*).

During 1943–57 X-raying rate in cancer cases increased as in controls but was always at a higher level. In 1958–61 it decreased abruptly nearly to control rates but by the 1970s had climbed to values as high as in 1954–7 (Figure 1). The abrupt decrease in case/control difference in 1957/8 might suggest an immediate reaction to the publications by Stewart *et al.* (1956, 1958) but, as will be seen, other factors are involved. The difference in X-raying rate between cases and controls (Tables VIII, IX and X) was in the direction expected if diagnostic X-rays are carcinogenic (except in 1962–5 and 1970–1 for cancer deaths at ages 6–9).

**Table VI** Proportions of X-rayed cases and controls by year of birth, ages 10–15 years 11 months

| Birth year | Case–control pairs | X-rayed children Cases % | X-rayed children Control % | Mean films per examination Cases | Mean films per examination Controls |
|---|---|---|---|---|---|
| 1940–41 | 22 | 4.5 | 18.2 | – | 1.0 |
| 1942–43 | 77 | 10.4 | 3.9 | 2.0 | 1.0 |
| 1944–45 | 142 | 6.3 | 7.0 | 1.0 | 1.0 |
| 1946–47 | 288 | 9.7 | 4.5 | 2.5 | 2.7 |
| 1948–49 | 350 | 11.4 | 6.3 | 1.6 | 1.8 |
| 1950–51 | 377 | 10.9 | 11.1 | 1.6 | 1.8 |
| 1952–53 | 348 | 18.7 | 13.8 | 1.8 | 2.1 |
| 1954–55 | 345 | 20.0 | 12.5 | 1.8 | 1.9 |
| 1956–57 | 304 | 14.5 | 13.1 | 1.8 | 2.2 |
| 1958–59 | 258 | 12.8 | 12.8 | 1.5 | 1.0 |
| 1960–61 | 271 | 11.4 | 11.1 | 1.4 | 1.1 |
| 1962–63 | 243 | 9.9 | 10.7 | 1.5 | 1.6 |
| 1964–65 | 148 | 15.5 | 8.1 | 1.0 | 1.0 |
| 1966–67 | 80 | 13.7 | 11.3 | 1.0 | 1.5 |
| 1968–69 | 8 | 0.0 | 12.5 | – | 1.0 |
| 1970–71 | – | – | – | – | 1.0 |
| 1972–73 | – | – | – | – | – |
| 1974–75 | – | – | – | – | – |
| 1976–77 | – | – | – | – | – |
| 1978 | – | – | – | – | – |
| Total | 3261 | 13.1 | 10.3 | 1.7 | 1.7 |

Data for cancer deaths at 10–15 years and their matched controls (Knox *et al.*, personal communication, 1989).

### *Number of X-ray films per X-ray examination*

The number of films per X-ray examination was used as a surrogate for magnitude of radiation dose when claiming that cancer rate increased progressively with increase in X-ray exposure (Stewart & Kneale, 1970*a*; Bithell & Stewart, 1975). It was based on a hospital's record and, when this did not exist, on an estimate by the hospital of the likely number of films that would have been exposed (Bithell & Stewart, 1975). Records and estimates were in the ratio 7:3 for both cancers and controls (Table 1, Kneale & Stewart, 1976*a*). Thus assessment of film number depended partly on an uncheckable recall of past events though not at all on a mother's memory. Kneale and Stewart (1976*b*) said the high proportion of pre-1960 deaths without a confirmed record 'was due partly to the absence of systematic recording of X-ray findings until completion of the pilot study of 1953–55 deaths [Stewart *et al.*, 1958] and partly to the inefficient recording of results of routine pelvimetries'.

Mean number of films per X-ray examination averaged 2.1–2.2 for 1948–57 and 1.3–1.4 subsequently (Table XIA). A similar decrease is seen in the late 1950s when birth years are grouped differently (Table XIB and C). Differences between cancer cases and their controls were small except for the earliest birth years 1940–7 (Table XIA). But these data can be no more than indicative since, as noted, film number per X-ray examination depended partly on an uncheckable recall of past events.

The case/control ratio of film number per X-ray examination is compared with the case/control ratio of X-raying rate in Figure 2 (the three age-at-death groups pooled). The former ratio was close to unity after 1940–7 (unexpectedly less than 1 after 1965). Film number seems less important for carcinogenesis than X-raying rate.

### *Reasons for X-raying*

In controls and cancer cases 14% and 17% of all obstetric X-ray examinations in birth years 1945–78 were pelvimetries (Gilman *et al.*, 1988). Pelvimetry was not mentioned specifically in a detailed cross-correlation of reasons for X-raying and the related findings (Kneale & Stewart, 1976*b*).

**Table VII** Radiation-discordant case/control pairs distributed by year of birth and age at death (showing number of pairs in which only the case (a) or only the control (b) was X-rayed)

| | *Age at death (years)* | | | | | | | | |
|---|---|---|---|---|---|---|---|---|---|
| | *0,1* | *2,3* | *4,5* | *6,7* | *8,9* | *10,11* | *12,13* | *14,15* | *Total* |
| *Year of birth* | *a/b* | *a/b* | *a/b* | *a/b* | *a/b* | *a/b* | *a/b* | *a/b* | *a/b* |
| 1940–3 | –/– | –/– | –/– | –/– | –/2 | 2/– | 4/3 | 2/1 | 8/6 |
| 1944–5 | –/– | –/– | –/– | –/3 | 5/3 | 2/1 | 3/2 | 4/5 | 14/14 |
| 1946–7 | –/– | –/– | 1/– | 8/1 | 12/2 | 7/1 | 5/2 | 11/9 | 44/15 |
| 1948–9 | –/– | 2/– | 14/7 | 16/4 | 17/11 | 8/2 | 16/8 | 11/6 | 84/38 |
| 1950–1 | 3/– | 17/16 | 22/9 | 23/12 | 19/5 | 11/10 | 11/12 | 10/13 | 116/77 |
| 1952–3 | 10/7 | 30/20 | 26/15 | 25/19 | 16/16 | 18/7 | 17/13 | 23/21 | 165/118 |
| 1954–5 | 17/17 | 41/22 | 41/19 | 28/20 | 23/19 | 20/10 | 17/14 | 20/7 | 207/128 |
| 1956–7 | 25/19 | 42/15 | 21/24 | 43/15 | 19/10 | 18/12 | 6/15 | 14/6 | 188/116 |
| 1958–9 | 24/14 | 35/27 | 20/21 | 26/20 | 18/12 | 12/10 | 5/9 | 11/9 | 151/122 |
| 1960–1 | 26/15 | 23/27 | 27/18 | 10/9 | 18/12 | 13/11 | 11/7 | 4/9 | 132/108 |
| 1962–3 | 24/16 | 29/28 | 29/16 | 14/21 | 8/14 | 8/5 | 7/10 | 4/4 | 123/114 |
| 1964–5 | 20/19 | 33/31 | 24/15 | 17/18 | 9/7 | 8/4 | 6/2 | –/– | 117/96 |
| 1966–7 | 27/26 | 24/15 | 17/15 | 12/13 | 7/13 | 4/4 | –/– | –/– | 91/86 |
| 1968–9 | 16/9 | 17/25 | 20/11 | 16/4 | 8/5 | –/– | –/– | –/– | 77/54 |
| 1970–1 | 27/15 | 21/17 | 21/11 | 8/8 | –/– | –/– | –/– | –/– | 77/51 |
| 1972–3 | 21/17 | 13/9 | 11/6 | –/– | –/– | –/– | –/– | –/– | 45/32 |
| 1974–6 | 25/13 | 4/5 | –/– | –/– | –/– | –/– | –/– | –/– | 29/18 |
| Total | 265/187 | 331/257 | 294/187 | 246/167 | 179/131 | 131/77 | 108/97 | 114/90 | 1668/1193 |

Data from Table 6 in Knox *et al.* (1987) with four additional discordant pairs; one control *b* for 1940–3 birth years, 14,15 years at death; one control *b* for 1944–5 birth years, 10,11 years at death; one control *b* for 1946–7 birth years, 6,7 years at death; and one case *a* for 1972–3 birth years, 2,3 years at death (Knox *et al.*, personal communication, 1989).

**Figure 1** Rate of X-raying *in utero* of future cancer cases and future matched controls in OSCC according to birth years 1940–1977, grouped as in Tables VIII, IX and X, and of other surveys in UK of the surviving children of women X-rayed when pregnant. The continuous line connects the grouped cohort-specific mean values for X-raying of cancer cases at all ages at death. The dashed line connects the grouped cohort-specific mean values for X-raying of controls matched for all ages at death. Individual mean values for controls matched to cancer cases dying at 0–5 years old Δ, at 6–9 years old O, and at 10–15 years old **X**. For birth years 1962–5 the X-raying rate of controls matched to cancer deaths at ages 6–9 years was 15% and at ages 0–5 years and 10–15 years was 12% and 10% respectively, the only statistically established difference in X-raying rate within any grouped birth cohort of controls. The rates for all ages are virtually the same as in Gilman *et al.* (1989*b*) except for cases in the 1970s where numbers of observations were larger and rates slightly smaller than in this figure. X-raying rate in pregnancy in other UK surveys: National Birthday Trust in 1958 (Stewart, 1973) and in 1970 (Dr J. Golding, personal communication, 1989) ●; Adrian Committee survey in Dec 1957 (rate from Kendall *et al.*, 1980) ▲.

X-raying for twins proved positive in 67% of future cases and 28% of future controls (620 cases, 473 controls). Only 1–2% of X-raying for other reasons revealed twins.

Stated reasons for X-raying over the 43 birth years 1939–81 were closely similar in future cases and future controls (Gilman *et al.*, 1989*b*), except for non-obstetric reasons in the early birth years 1939–49 (11% of all reasons in cases, 2% in controls). X-raying for twins averaged about one in three of all examinations over the 43 year period. Fetal maturity, as a reason, increased strikingly from 1–2% in 1939–49 to 8–11% in 1960–9 and then to 25–26% in 1970–81, and absence of a recorded reason decreased from about 50% to 25%. These data concern reasons for X-rays, not findings.

*Odds ratio for cancer: radiation-discordant case/control pairs*

The odds ratio (OR) is the number of paired X-rayed cancer cases with matched but not X-rayed controls divided by the number of paired cancer cases not X-rayed and with matched controls who were X-rayed (Table VII). Differences in OR for different ages at death were small within each birth year grouping (Table XIII). In each age-at-death group OR was higher for births before 1958 than for births after 1957, significantly so for ages 6–9 years and for all ages pooled (Table XIII). OR for birth years 1958–65 and 1966–9 was the same. Something occurring about 1957/8 reduced cancer risk after prenatal exposure to X-rays. When all ages at death are pooled, OR for the 4 year birth cohorts 1953–7 = 1.62 (90% CI 1.40–1.87) and 1958–61 = 1.23 (90% CI 1.05–1.44).

OR values for 1940–7 are the largest but also have the widest confidence intervals (Table XIII). If data from these war and immediate post-war years are accepted as valid, then some reduction in the effect of X-raying may have occurred long before attention was drawn to the cancer risk by the first publications of Stewart *et al.* (1956, 1958). The progressive decrease in relative risk with calendar year of birth in Figure 2 in Bithell and Stewart (1975) depended largely on the inclusion of births in 1940–7.

*Matching of controls by place of birth as well as place of death.* When cases and controls share a common birth year, the mean intrauterine radiation dose is likely to have been similar, independently of systematic changes in fetal dose per X-ray examination over the years 1940–77. Some 16–21% of cancer cases moved to a new administrative district between birth and death (Knox *et al.*, 1987). Thus for 79–84% of case/control pairs the matching of cases and controls was by place (civil district) of birth, as well as of death, implying that the circumstances of X-raying before birth were usually similar, especially since the commonest time of prenatal X-raying is shortly before birth (Table XIV). When range of fetal dose and its mean are similar, the ratio of X-raying rate in cancer cases to that in matched controls is a simple measure of relative risk for carcinogenesis by X-rays that is insensitive to temporal changes in specific magnitude of fetal dose per examination. This ratio

**Table VIII** Case/control pairs aged 0–5 years 11 months: number, odds ratio, X-raying rate and number of films per X-ray examination according to year of birth

| Cohort birth years | Case/control pairs: Number | Case/control pairs: Radiation-discordant a/b[a] | Case/control pairs: Missing information[b] | Odds ratio: Birth cohort (90% confidence intervals) | Odds ratio: Grouped average[c] (90% confidence intervals) | X-raying rate: Case % | X-raying rate: Control % | X-raying rate: Ratio | Film number per X-ray examination: Birth cohort average: Case | Birth cohort average: Control | Grouped average[c]: Case | Grouped average[c]: Control |
|---|---|---|---|---|---|---|---|---|---|---|---|---|
| 1946–7 | 25 | 1/0 | | | | 8 | 4 | | 3.0 | | | |
| 1948–53 | 1428 | 124/74 | 15/42 | 1.68 (1.27–2.21) | 1.64 (1.39–1.93) | 15.8 | 10.4 | 1.52 | 2.51 | 2.30 | 2.3 | 2.2 |
| 1954–57 | 1332 | 187/116 | | 1.61 (1.30–2.00) | | 19.1 | 12.2 | 1.56 | 2.20 | 2.09 | | |
| 1958–61 | 1508 | 155/122 | | 1.27 (1.02–1.59) | | 12.0 | 9.5 | 1.27 | 1.75 | 1.60 | | |
| 1962–5 | 1352 | 159/125 | | 1.27 (1.02–1.58) | 1.25 (1.10–1.42) | 13.9 | 11.5 | 1.21 | 1.60 | 1.30 | 1.6 | 1.4 |
| 1966–9 | 1041 | 121/101 | | 1.20 (0.93–1.54) | | 14.0 | 11.9 | 1.18 | 1.39 | 1.45 | | |
| 1970–3 | 727 | 114/75 | 1/28 | 1.52 (1.15–2.01) | | 20.9 | 15.5 | 1.35 | 1.24 | 1.35 | 1.2 | 1.4 |
| 1974–6 | 223 | 29/18 | 9/21 | 1.61 (0.95–2.79) | | [d] 19.9 | 13.9 | 1.43 | 1.12 | 1.46 | | |

[a]a/b number of pairs in which only the case (a) or only the control (b) was X-rayed. [b]Number of cells without information/number of cells in the complete cohort (Table I). [c]Based on combined data. An average is not given for 1970–6 because so much information for 1974–6 if not yet available. [d]X-raying rate and film number per X-ray examination for 1974–7 (251 case/control pairs Table IV).

**Table IX** Case/control pairs aged 6–9 years 11 months: number, odds ratio, X-raying rate and number of films per X-ray examination according to year of birth

| Cohort birth years | Case/control pairs: Number | Case/control pairs: Radiation-discordant a/b[a] | Case/control pairs: Missing information[b] | Odds ratio: Birth cohort (90% confidence intervals) | Odds ratio: Grouped average[c] (90% confidence intervals) | X-raying rate: Case % | X-raying rate: Control % | X-raying rate: Ratio | Film number per X-ray examination: Birth cohort average: Case | Birth cohort average: Control | Grouped average[c]: Case | Grouped average[c]: Control |
|---|---|---|---|---|---|---|---|---|---|---|---|---|
| 1943–7 | 430 | 25/11 | 10/25 | 2.27 (1.20–4.50) | | 12.3 | 4.2 | 2.94 | 3.1 | 1.6 | | |
| 1948–53 | 970 | 116/67 | | 1.73 (1.29–2.32) | 1.75 (1.43–2.13) | 16.0 | 9.5 | 1.68 | 2.6 | 2.3 | 2.4 | 2.1 |
| 1954–57 | 604 | 113/64 | | 1.77 (1.31–2.38) | | 22.7 | 14.7 | 1.54 | 2.2 | 2.0 | | |
| 1958–61 | 588 | 72/53 | | 1.36 (1.08–1.75) | | 13.4 | 10.5 | 1.27 | 1.7 | 1.3 | | |
| 1962–5 | 471 | 48/60 | | 0.80 (0.61–1.12) | 1.10 (0.89–1.36) | 12.7 | 15.3 | 0.83 | 1.2 | 1.4 | 1.4 | 1.4 |
| 1966–9 | 388 | 43/35 | 1/20 | 1.23 (0.82–1.84) | | 13.4 | 11.6 | 1.16 | 1.3 | 1.5 | | |
| 1970–1 | 101 | 8/8 | 5/10 | | | [d] 15.7 | 18.5 | 0.85 | 1.2 | 1.4 | | |

[a]a/b number of pairs in which only the case (a) or only the control (b) was X-rayed. [b]Number of cells without information/number of cells in the complete cohort (Table II), [c]Based on combined data. [d]X-raying rate and film number per X-ray examination for 1970–3 (108 case/control pairs Table V).

**Table X** Case/control pairs aged 10–15 years 11 months: number, odds ratio, X-raying rate and number of films per X-ray examination according to year of birth

| Cohort birth years | Case/control pairs: Number | Case/control pairs: Radiation-discordant a/b[a] | Case/control pairs: Missing information[b] | Odds ratio: Birth cohort (90% confidence intervals) | Odds ratio: Grouped average[d] (90% confidence intervals) | X-raying rate: Case % | X-raying rate: Control % | X-raying rate: Ratio | Film number per X-ray examination: Birth cohort average: Case | Birth cohort average: Control | Grouped average[d]: Case | Grouped average[d]: Control |
|---|---|---|---|---|---|---|---|---|---|---|---|---|
| 1940–45 | 241 | 17/12 | | 1.4 | | 7.5 | 7.1 | | 1.5 | 1.0 | | |
| 1946–7 | 288 | 23/12 | | 1.9 | | 9.7 | 4.5 | | 2.5 | 2.7 | | |
| 1940–7 | 529 | 40/24 | 15/56[c] | 1.67 (1.06–2.65) | | 8.7 | 5.7 | 1.53 | 2.1 | 1.7 | | |
| 1948–53 | 1075 | 125/92 | | 1.36 (1.05–1.76) | 1.41 (1.17–1.70) | 13.6 | 10.4 | 1.30 | 1.7 | 1.9 | 1.7 | 2.0 |
| 1954–7 | 649 | 95/64 | | 1.48 (1.09–2.03) | | 17.4 | 12.8 | 1.36 | 1.8 | 2.0 | | |
| 1958–61 | 529 | 56/55 | | 1.02 (0.73–1.42) | 1.11 (0.83–1.49) | 12.1 | 11.9 | 1.02 | 1.5 | 1.0 | 1.4 | 1.2 |
| 1962–5 | 391 | 33/25 | 6/28 | 1.32 (0.83–2.12) | | 12.0 | 9.7 | 1.24 | 1.3 | 1.4 | | |
| 1966–7 | 80 | 4/4 | 15/21 | | | [e] 12.5 | 11.4 | 1.10 | 1.0 | 1.5 | | |

[a]a/b number of pairs in which only the case (a) or only the control (b) was X-rayed. [b]Number of cells without information/number of cells in the complete cohort (Table III), [c]Birth years 1940–43 15 empty cells/28; birth years 1944–45 no empty cells/14 but number of cancers per birth year killing at 10–15 years of age was about 40% of that in the succeeding birth years 1946–57 (cf. text and Table III), [d]Based on combined data. [e]X-raying rate and film number per examination for 1966–9 (88 case/control pairs Table VI).

**Table XI** Number of films per X-ray examination in cancer cases and in their matched controls according to calendar years of birth and the age at death of the cancer cases

| | *Age at death of cancer cases* | *Film number per examination in birth year cohorts* | | | |
|---|---|---|---|---|---|
| A[a] | | *1940–7* | *1948–57* | *1958–65* | *1966–9* |
| *Cancer cases* | | | | | |
| | 0–5 years 11 months | | 2.3 | 1.7 | 1.4 |
| | 6–9 years 11 months | 3.1 | 2.4 | 1.5 | 1.3 |
| | 10–15 years 11 months | 2.1 | 1.7 | 1.4 | (1.0)[b] |
| | All ages | 2.6 | 2.2 | 1.6 | 1.3 |
| *Matched controls* | | | | | |
| | 0–5 years 11 months | | 2.2 | 1.4 | 1.5 |
| | 6–9 years 11 months | 1.6 | 2.1 | 1.3 | 1.5 |
| | 10–15 years 11 months | 1.7 | 2.0 | 1.2 | (1.5)[b] |
| | All ages | 1.7 | 2.1 | 1.4 | 1.5 |
| B[c] | | *1943–9* | *1950–4* | *1955–9* | *1960–5* |
| Cases | all ages | 2.5 | 2.4 | 2.0 | 1.6 |
| Controls | all ages | 1.9 | 2.2 | 1.9 | 1.5 |
| C[d] | | *1939–49* | *1950–9* | *1960–9* | *1970–81* |
| Controls | all ages | 1.77 | 2.10 | 1.39 | 1.39 |

[a]From Tables VIII, IX and X. [b]Values based on very small numbers (Table VI). [c]Derived from Table 1 in Stewart & Kneale (1970*a*). [d]For dated X-rayings (Table 3, Gilman *et al.*, 1989*b*).

**Figure 2** Cancer case/control ratios for rate of X-raying and for film number per X-ray examination according to birth years 1940–1977, grouped as in Tables VIII, IX and X, for all childhood cancer deaths at ages 0–15 years and their matched controls. X-raying per cent case/control ■——■; Film number per X-ray examination case/control □– –□.

**Table XII** Mantel–Haenszel estimates of relative risk for X-rays classified by trimester and number of films

| *X-ray specifications* | | *Relative risk*[a] | |
|---|---|---|---|
| Exposure date | First trimester | 2.69 | |
| | Second trimester | 0.91 | |
| | Third trimester | 1.00 | $\chi^2_{(3)} = 9.05$ |
| | No record | 1.01 | $0.025 < P < 0.05$ |
| Number of films | 1 | 1.00 | |
| | 2 | 1.08 | |
| | 3 | 0.97 | $\chi^2_{(5)} = 1.83$ |
| | 4 | 1.07 | |
| | 5+ | 1.18 | |
| | No record | 0.94 | Not significant |

[a]Controlling factors: sex, birth year, social class, maternal age, sibship position, also exposure-age or film number (see text). The relative risk for each test factor level is compared with the factor level setting the standard (i.e. RR = 1.00). Table 2, Gilman *et al.* (1988). The information on intrauterine age at exposure and on film number was all obtained from medical records and is not dependent on a mother's recollection of events. It came from 58% of both cases and controls but not exclusively from case/control pairs.

decreased from 1954–7 to 1958–61 in each of Tables VIII, IX and X.

*Other carcinogenic influences in pregnancy*

Many possibly causal factors have been isolated by conditional logistic regression applied to case/control pairs in the OSCC (Gilman *et al.*, 1989*a*). The dates when specific drugs were introduced or became commonly used, such as pethidine for pain relief in labour, or when specific procedures were abandoned, such as vaccination against smallpox, were not taken into account. The OSCC did not begin to record the use of drugs in pregnancy until 1964 (Knox *et al.*, 1987). In this review of data from death years 1953 onwards I have assumed that paired cases and controls were likely to be more like each other for other possible causal factors in a group of 4–6 consecutive birth years than for all birth years 1940–76 pooled.

It was reported (Knox *et al.*, 1987; Gilman *et al.*, 1989*a*) that RR for irradiation in the OSCC data increased when the carcinogenic influence of other factors in pregnancy (maternal illnesses, drugs etc) were allowed for but this was the result of an error in statistical inference (Muirhead & Kneale, 1989). Nevertheless, in the OSCC birth cohorts for 1964–79 the carcinogenic 'effect of X-rays is certainly not reduced by controlling for illnesses and drugs' (loc. cit.).

### Aspects of obstetric radiography

*X-raying rate during pregnancy: surveys other than the OSCC*

In England in 1973–4 the abdominal X-raying rates in pregnancy were 8.6% and 16.5% in two, and 23–35% in six, of eight major hospital maternity centres (Carmichael & Berry, 1976). Rates 23–35% are unexpectedly higher than the mean rate for the 1970s in OSCC matched controls (Figure 1). In both the rate is for X-raying of mothers (not of fetuses). (Matched OSCC controls sometimes included one of a pair of twins but never both). Thus X-raying rate could differ widely between different localities. The lowest rate, 8.6%, came from a centre without its own X-ray equipment.

The only reference to X-raying in pregnancy in a national survey of births in 1946 is a note that pelvic X-ray measurements were made at the thirty-second week in nearly all primigravidae in Kent (Joint Committee, 1948). Some post-war clinics used pelvimetry as a routine in primigravidae (Browne, 1951). The nationwide rate of obstetric radiography in 1957 was 11.4% in live births (Kendall *et al.*, 1980).

In 1958 and 1970 a longitudinal study of all births in one week in Great Britain was organised by the National Birthday Trust NBT (Butler & Bonham, 1963; Chamberlain *et al.*, 1978). Soon after each birth its circumstances were noted from contemporary records: X-raying in pregnancy was a

**Table XIII** Odds ratios with 90% confidence intervals for X-raying of cancer cases *vs* matched controls according to calendar years of birth and age at death of the cancer cases

| | Birth year cohorts | | | | | | | |
|---|---|---|---|---|---|---|---|---|
| | *1940–7* | | *1948–57* | | *1958–65* | | *1966–9* | |
| *Age at death* | *OR* | *90% CI*[a] | *OR* | *90% CI* | *OR* | *90% CI* | *OR* | *90% CI* |
| 0–5 years 11 months | | | 1.64 | 1.39–1.93 | 1.27 | 1.09–1.93 | 1.20 | 0.93–1.54 |
| 6–9 years 11 months | 2.27 | 1.20–4.50 | 1.75 | 1.43–2.13[b] | 1.06 | 0.83–1.36[b] | 1.23 | 0.82–1.84 |
| 10–15 years 11 months | 1.67 | 1.06–2.65 | 1.41 | 1.17–1.70 | 1.11 | 0.83–1.49 | | |
| All ages | 1.86 | 1.40–2.55 | 1.59 | 1.44–1.76[b,c] | 1.19 | 1.06–1.33[b,c,d] | 1.21 | 0.97–1.49[d] |

[a]OR 90% CI = odds ratio with 90% confidence intervals. [b]90% confidence intervals for birth years 1948–57 and 1958–65 do not overlap. [c]95% confidence intervals do not overlap, 1.41–1.80 and 1.05–1.35 respectively. [d]OR for pooled birth years 1958–69 = 1.19 (90% CI 1.08–1.31).

**Table XIV** Dated X-rays in pregnancy at different stages of intra-uterine development: mothers of future childhood cancers and future matched controls (Table 8.2, Mole, 1989)[a,b]

| | | Number of abdominal and pelvic X-ray exposures | | | | | |
|---|---|---|---|---|---|---|---|
| | | Obstetric examinations | | Non-obstetric examinations | | | |
| *Age post-conception (completed weeks)*[c] | | | | *All*[d] | | *With fluoroscopy*[e] | |
| | | *Cancer* | *Control* | *Cancer* | *Control* | *Cancer* | *Control* |
| Conceptus and embryo | 0–7 | 3 | 3 | 22 | 5 | 7 | 3 |
| Fetal I | 8–24 | 70 | 48 | 27 | 17 | 8 | 5 |
| Fetal II | 25–38 | 1299 | 996 | 18 | 18 | 1 | 1 |

[a]Partition of fluoroscopic examinations between conceptus and embryo and fetal stage I was incorrect in Table 8.2, Mole (1989). [b]X-rays confirmed by medical records in 1944–78 of 1,439 future cancers and 1,087 future controls (Table 5, Gilman *et al.*, 1988), excluding four cancers and one control X-rayed more than 38 weeks before delivery (Knox *et al.*, personal communication, 1989). Cases and controls were not matched pairs. [c]Age post-conception (completed weeks) at time of X-raying = 38 weeks less interval between X-ray date and birth date (weeks), 38 completed weeks from oocyte fertilisation to birth day is the 'standard' duration of normal development *in utero*. Conceptus and embryo are within the first trimester. Fetal I corresponds to last month of first trimester plus second trimester, fetal II to third trimester. [d]Only in 1939–49 were non-obstetric X-rays much more frequent in future cancer cases than future controls, 10.5 and 1.9%, but 4.9 and 4.1% in 1950–81 (Table 4, Gilman *et al.*, 1989*b*). [e]Examinations using contrast medium (but excluding pyelography) from Table 3 (Gilman *et al.*, 1988), with additional information about numbers of cases and controls in my categories conceptus and embryo and fetal I (Knox *et al.*, personal communication, 1989).

specific datum to be recorded. 1958 NBT data for survivors at one month after delivery gave an X-raying rate 10.7% for singletons and 11.6% for all children, singletons and twins (Stewart, 1973). The corresponding rates in OSCC controls (mothers) for singleton births in 1958–9 were 10.6% overall, and 9.3%, 11.8% and 12.8% for controls matched for cancer deaths at ages 0–5, 6–9 and 10–15 years (data in Tables IV, V and VI).

The 1970 NBT cohort of neonatal survivors had an X-raying rate of abdomen and/or pelvis for all mothers 2045/16357 (Dr J. Golding, personal communication, 1989) = 12.5% (s.e. 0.26%), for mothers of singletons 11.9% and of twins 73%. The 1970–1 rate in OSCC matched controls 79/515 (Tables IV, V and VI) = 15.3% (s.e. 1.6%) and in all OSCC controls 94/629 (Table I, Gilman *et al.*, 1989*b*) = 14.9% (s.e. 1.4%). The respective $t$ values for excess above the 12.5% NBT rate are 1.8 and 1.7 ($P = 0.05–0.1$).

If rates of radiography vary substantially between civil districts and if X-ray exposure does induce cancer in the fetus, then the OSCC method for selection of matched controls will give a control X-raying rate larger than the true population rate. There must be (on average) a higher childhood cancer rate in children born in localities with relatively high X-raying rates in pregnancy than in localities with relatively low X-raying rates. Thus controls with the same place of birth as cancer cases will come more often than randomly from localities with higher X-raying rates and less often than randomly from localities with relatively low X-raying rates. But the data in the previous two paragraphs show that the method of selection of OSCC controls did not cause large deviations of OSCC rate from the true X-raying rate either in 1958 or 1970–1.

The low obstetric X-raying in pregnancy in 1978–9 reported by Kendall *et al.* (1980) underestimated it by 2–3 times (Kendall *et al.*, 1989). By the late 1970s ultrasound was commonly used for fetal surveillance but had hardly influenced the X-raying rate in pregnancy (Gilman *et al.*, 1989*b*).

### *The Adrian Committee survey of radiological practice 1956–8*

When planning to estimate population gonad dose originating in medical radiology the Adrian Committee needed to know the number of each type of X-ray examination carried out per year and the associated specific gonad dose. Preliminary estimates of numbers and types for 1955 were based on a small sample of hospitals. Later a questionnaire asked all NHS hospitals, clinics etc to record the number of X-ray examinations of different types in a specified week in April/May 1957. A second questionnaire sent to a random 25% sample of NHS hospitals stratified by size asked for similar but not identical information for a specified week in December 1957.

In December as compared with May 1957 the reported X-raying rate was smaller by 30% for obstetric abdomen examinations and by 50% for pelvimetry (Table XV). 'The impression was left that by mid-1958, when the [dose] measurements were made, there had been a further decrease, although there is no firm evidence in support of this. Several hospitals reported that they no longer carried out such examinations while others had reduced their numbers drastically, in some cases to 10% or less of the pre-1956 figure.'

**Table XV** Estimated number of obstetric radiological examinations per year in England and Wales (from Table 45 and text of Osborn, 1960)

| | | *Obstetric abdomen* | *Pelvimetry* |
|---|---|---|---|
| Inferred from a sample of 10 hospitals | 1955 | at least 86,000 | at least 26,000 |
| Response to questionnaire[a] | May 1957 | 82,000 | 26,000 |
| Response to questionnaire[a] | Dec 1957 | 59,000 | 14,000 |

[a]Number in a specified week × 52 and adjusted to exclude Scotland.

(p. 104, Osborn, 1960). Even before measurements were made the contribution of pelvimetry to gonad dose was declining (Spiers, 1957). Thus reduction in radiography for obstetric purposes seems adequate to explain the specific shortfall in dose measurements for obstetric but not other X-ray examinations. The numbers asked for were based on the May 1957 questionnaire but in 1958 only 32% of requested measurements for pelvimetry were actually made, as compared with 121% for chest X-rays and 65–90% for other kinds of examination (Appendix 1 Table 7, Ministry of Health, 1960).

*Dose to the ovary in general medical radiology.* The magnitude of ovary dose in an X-ray examination is some indication of level of intrauterine dose. Ovary dose in diagnostic X-ray examinations varied widely between different hospitals and even within single departments of radiology. One or two hospitals did not restrict the X-ray beam, which could be up to a measured 1.3 m in diameter (Osborn, 1961). Few X-ray sets were equipped with a light beam diaphragm allowing the X-ray beam to be restricted to the useful area of an X-ray film. Ovary dose ranged from maximum values in the direct beam to minimum values when the beam area did not exceed that of the film being exposed and the only radiation reaching the ovary was from scatter within the body of the woman. Consequently the range for chest, heart and lung X-rays was nearly 5 orders of magnitude from 0.01 to 500 mR (Figure 5A, Appendix 1, Ministry of Health, 1960) and dose distribution was highly skewed in each case. (Note that in this review of effects of diagnostic X-rays the radiation dose is stated in the same units as in the original publications. For my purposes R, rad and cGy are taken as interchangeable.)

In women in 1958 the ovary was in the direct beam in over 50% of routine large film X-rays of the chest and in 9% of X-ray examinations of arm and hand (Osborn, 1963). Mean ovary dose was 2.2 and 3.6 mR for X-rays of arm, hand and of leg, foot (Matthews & Miller, 1969), about half the mean ovary dose 5.4 mR per examination for chest X-rays (Table 1, Appendix 1, Ministry of Health, 1960). The assertion that X-raying of chest and extremities would have no effect on the fetus *in utero* (Kneale & Stewart, 1980), taken literally, seems not to apply before 1959.

Limiting an X-ray beam to the useful area of a film was the major Adrian Committee recommendation for reducing gonad dose in all radiological examinations (Ministry of Health, 1960). By 1964 mean ovary dose in adult women receiving a chest X-ray in the Sheffield Region had been reduced 26-fold from 5.5 to 0.21 mR per examination (Matthews & Miller, 1969). In 1978–9 a nationwide value was 0.2 mrad (Wall *et al.*, 1980), no smaller than 15 years earlier in the Sheffield region.

*Obstetric X-ray examinations and fetal radiation dose* An obstetric abdomen X-ray is intended to image the whole fetus. A large film is used and the fetus will be more-or-less uniformly irradiated. In pelvimetry the aim is to show the bony structure of the maternal pelvis and the part of the fetus within the pelvis at the time. Details of projection and technique determine how much of the fetal body and gonads are in the direct beam and how much is exposed only to scattered radiation. Occasionally pelvimetry is needed in a non-pregnant woman who has recently had a difficult labour and needs advice about future pregnancies.

In 1958 the range of maternal ovary and fetal gonad dose in obstetric abdomen and pelivimetry examinations was about two orders of magnitude (Figures 5E and F, Appendix 1, Ministry of Health, 1960), much smaller than the five orders of magnitude for maternal ovary dose in other diagnostic examinations. In obstetric abdomen examinations the maternal ovary is in the direct beam, scattered radiation is relatively unimportant, mean dose is much larger and dose distribution is much less skewed.

Mean fetal gonad dose in different pelvimetric projections differed by up to 16-fold (Table XVI). Thus fetal dose in pelvimetry cannot be assessed without specific knowledge of the projections used. If Thoms' view is omitted (as strongly recommended by Blair Hartley, 1956) and a four projection pelvimetry is replaced by a three (or two) projection examination, the total fetal gonad dose is reduced by 2.5–3 times (or more), from about 3,500 to about 1,300 mR (or less) (Table XVI). Thoms' projection must have been rarely, if ever, used in 1958 when mean fetal gonad dose for pelvimetry was 885 mR (Table I, Ministry of Health, 1960).

Mean fetal gonad dose for the same pelvimetry projections differed 5-fold in two London teaching hospitals (Osborn, 1951; Stanford, 1951). A report from a specialist maternity hospital in London (Martin & Williams, 1946) indicates that doses in earlier years could sometimes have been as low as for good techniques in the late 1950s, confirming that fetal dose in pelvimetry varied widely during the years before 1958.

*Change in practice and meaning of 'pelvimetry' in 1957–8.* Pelvimetry was being developed during the decade before the Adrian survey. Seven techniques were described in a major British textbook on X-ray diagnosis (Williams, 1950). Each was intended to give information about mechanical aspects of delivery, the dimensions of the head of the fetus (its largest part) and of the birth canal within the maternal pelvis through which the fetal head must pass. In a standard British textbook on antenatal care Moir wrote (1951, 1955): 'The practical value of X-ray pelvimetry is now generally agreed by both obstetricians and radiologists. . . . Hitherto, radiologists have been feeling their way with these new methods of investigation, but now, with better techniques, and better methods of interpretation of the radiographic findings, they can give much firmer guidance to the obstetrician.' Before the Adrian survey methodology in pelvimetry was not standardised and consequently fetal dose was not standardised either.

**Table XVI** Mean fetal and maternal gonad dose in pelvimetry according to projection (Table VII, Ministry of Health 1960)

| | *Mean gonad dose mR per exposure* | |
|---|---|---|
| *Projection* | *Maternal ovary* | *Fetal gonad* |
| 1. Antero-posterior | 460 | 630 |
| 2. Lateral | 577 | 535 |
| 3. Sub-pubic arch and pelvic outlet | 670 | 140 |
| 4. Supero-inferior, pelvic inlet or Thoms'[a,b] | 992 | 2,242 |

All four projections were described as routine examinations in Clark (1956). In the next edition Clark (1964) said about pelvimetry in pregnant women 'only two projections are used', projection no. 4 'can no longer be tolerated' and no. 3 'is not a routine'. [a]Also termed antero-posterior oblique 'brim view' (e.g. Clayton *et al.*, 1957). [b]Projection no. 4 was abandoned as a routine procedure because of concern over the magnitude of the associated fetal gonad dose, even when using Moir's method which ensured that the fetal gonads were usually outside the direct X-ray beam. Moir (1960) wrote that the pelvic inlet view 'cannot be recommended for the woman near term. Clear pictures are not possible'. This difficulty is unavoidable when the bulky gravid uterus is interposed between the X-ray tube focus and the maternal pelvis (c.f. Figure 6 and 7, Clayton *et al.*, 1957).

During 1958 a single lateral exposure of the pelvis tended to be described as a pelvimetric examination (the late Professor R.E. Ellis, personal communication, 1963). It came to be accepted that in a great majority of cases a single lateral view of the pelvis (projection 2, Table XVI) interpreted by an experienced radiologist would meet the needs of an obstetrician concerned with possible disproportion between a baby's head and the space within the pelvis necessary for safe delivery (Dr J.H.E. Carmichael, personal communication, 1989). Previously a routine pelvimetry always involved multiple films, at least one for each of two, three or more projections (Moir 1951, 1955; Williams, 1950; Table XVI). In 110 measured pelvimetric examinations in 1958 (Ministry of Health, 1960), 69 used only a single film, of which 63 were lateral projections (Table XVII).

The 1964 edition of a widely used medical radiographer's bench book said about pelvimetry, 'one or two projections are used [cf. Table XVI]: the pelvic inlet projection [Thoms'] can no longer be tolerated as a routine . . . the view of the pelvic outlet is not a routine' (Clark, 1964). The 1956 forecast was fulfilled: 'In the light of current pronouncements on genetic hazards it is likely that X-ray examination of the pregnant subject will be drastically restricted in the near future' (Clark, 1956).

*Change in film number per X-ray examination in pregnancy.* An abrupt decrease in film number per examination in the late 1950s is confirmed in Adrian survey data. Film number per examination for pelvimetry was up to nine and not less than three in seven different hospitals in 1955/6 (Osborn & Smith, 1956). In December 1957 mean number was 2.0 for pelvimetries and 1.3 for obstetric abdomen examinations (Table 4, Appendix 1, Ministry of Health, 1960). At the measured examinations some months later in 1958, mean film number was 1.7 (or 1.53, Table XVII) for pelvimetry and 1.2 for obstetric abdomen X-rays (Table 11, Appendix 1, Ministry of Health, 1960). In a limited survey in 1978–9 film number was 1.0 and 1.2 respectively (Wall *et al.*, 1980).

**Table XVII** Average fetal whole body dose[a] per examination and per X-ray film in pelvimetric and obstetric abdomen examinations in 1958 in Britain (from unpublished data collected by the Adrian survey: the late Professor R.E. Ellis[b], personal communication, 1963).

| | *Film number per examination* | *Number of examinations* | *Average whole body dose R per examination* | *Average whole body dose R per film* | |
|---|---|---|---|---|---|
| Pelvimetry | 1 | 69[c] | 1.11 | 1.11 | |
| | 2 | 28 | 0.94 | 0.47 | } 0.47 |
| | 3 | 9 | 1.49 | 0.50 | |
| | 4 | 4 | 1.69 | 0.52 | |
| All[d] | 1.53[e] | 110 | 1.12[f] | | |
| Obstetric | 1 | 90 | 0.40 | 0.40 | |
| Abdomen | 2 | 21 | 0.89 | 0.45 | |
| All[g] | 3 | 1 | 0.92 | 0.31 | |
| | 1.21 | 112 | 0.50[h] | 0.41 | |

[a]Observations by Bewley *et al.* (1957) and Clayton *et al.* (1957) provided factors allowing average fetal dose per unit maternal skin dose to be deduced for each projection and each X-ray quality used in the X-ray examinations of the Adrian survey. This factor multiplied by the maternal skin dose measured in a particular examination gave the whole body fetal dose for that examination. Marrow dose in a fetus was taken to be the same as its whole body dose. [b]formerly Secretary, Panel of Physicists, Adrian Committee. [c]63 of the 69 consisted solely of a single lateral 'view'. The tendency to use one lateral film only in examinations termed 'pelvimetry' seemed to increase dose per film as compared with examinations using several films. [d]11 Inlet, 21 Outlet, 102 Lateral, 22 A-P, 12 P-A. [e]1.53 (Ellis, personal communication) is not the same as 1.7 in the Adrian Committee Report (Table II, Ministry of Health, 1960) for unknown reasons. [f]In hospitals with more than 300 beds and less than 300 beds average dose was 0.81 R in 73 examinations and 1.7 in 37 examinations (ranges 0.13–4.9 R and 0.17–3.5 R) respectively. Mean number of films per examinations was 1.3 and 1.9. [g]58 A-P, 51 P-A, 22 Lateral. [h]In hospitals with more than 300 beds and less than 300 beds average dose was 0.47 R in 73 examinations and 0.55 R in 39 examinations (ranges 0.03–2.1 R and 0.03–2.0 R) respectively.

*Six years after the Adrian Committee investigations*

The Sheffield Hospital Region had been the most successful of all in 1958, determining dose in 18% more examinations than requested (Osborn, 1960). Six years later in 1964 population gonad dose was re-assessed using the same methods and on a larger scale (Matthews & Miller, 1969). Mean fetal gonad dose per pelvimetry was 710 mR, close to the 1958 national average, and for obstetric abdomen examinations was 203 mR, much smaller than the 1958 national average 720 mR. A reason for this substantial decrease was not given. Film numbers per examination for different kinds of non-obstetric X-ray examinations were similar to the 1958 national averages.

*Twenty years after: a National Radiological Protection Board survey*

During the years after 1958 radiation dose should have decreased as a result of technical changes in diagnostic radiology, including faster films and rare earth screens. Measurements in a limited survey in 1978/9 (Wall *et al.*, 1980) showed some reduction in dose, by 50% for fetal gonad dose in obstetric abdomen examinations. (The term 'fetal maturity' in Wall *et al.* (1980) is synonymous with obstetric abdomen: Dr S. Rae, personal communication, 1989). Rare earth screens were used in 70% of the obstetric dose determinations but for general obstetric work in only five of 21 hospitals surveyed. Thus the measured fetal dose in 1978/9 will overestimate the nationwide dose reduction since 1958. Mean fetal gonad dose from an obstetric abdomen X-ray was 347 cGy, larger than 203 mR, the 1964 value of Matthews and Miller (1969).

### Determination of intrauterine dose in obstetric radiography

Intrauterine (and ovary) dose cannot be measured *in vivo* but only in phantoms with the physical dimensions of a pregnant woman's abdomen. Dose can be measured on the abdominal surface of a phantom and at the corresponding points *in vivo* on the abdomen of a pregnant woman. Inferences can then be made about the intrauterine (and ovary) dose *in vivo* using scaling factors, derived from direct knowledge of the position of measuring devices within a phantom, and assumptions about the detailed geometry of the position of uterus and fetus within the pregnant abdomen *in vivo*. The scaling factors will vary with the conditions of irradiation, such as X-ray kilovoltage and filtration, distance of X-ray tube focus from the abdominal surface and the X-ray film, etc. Scaling factors were derived by Bewley *et al.* (1957) and Clayton *et al.* (1957) and all assessments of fetal dose and of maternal gonad dose in Adrian survey reports (Ministry of Health, 1960, 1966) were based on their work. Each investigation dealt in detail with dose from different pelvimetric views, seven in Clayton *et al.* (1957), three in Bewley *et al.* (1957), and the latter also gave fetal and maternal doses for lateral and PA obstetric abdomen examinations.

During early pregnancy the gonads of embryo or fetus within the uterus lie near the maternal ovaries. During late pregnancy, when most obstetric radiography is done, the distance between them increases as the maternal ovaries are pushed cephalad by the expanding uterus. Thus the relationship of dose in maternal ovary and in fetus changes with stage of pregnancy.

The Final Adrian Committee Report gave estimates of mean whole-body fetal dose from pelvimetry and from obstetric abdomen examinations made in 1958 (Table II, Ministry of Health, 1966). These referred to late pregnancy when 90–95% of all these examinations are made. Whole body dose was taken to be an estimate of marrow (haematopoietic tissue) dose and thus of the relevant dose for induction of all childhood cancer, including leukaemia.

Fetal gonad and whole body dose may be very different (Table XVIII). Their ratio varied from 0.2 to 3.5 for three

**Table XVIII** Fetal gonad and whole body dose from obstetric X-ray examinations in late pregnancy

| | | *Mean fetal gonad dose (R)* | | *Fetal whole body dose (R)* | *Maternal gonad dose mean (R)* |
|---|---|---|---|---|---|
| *From Bewley et al. (1957) assuming specific conditions for radiography* | | | | | |
| | | a | b | c | a |
| Pelvimetry projection | No. 2 Lateral | 0.06, 0.14 | 0.1 | 0.3 | 0.1 |
| | No. 3 Outlet | 0.01, 0.02 | 0.02 | 0.1 | 0.03 |
| | No. 4 Inlet | 4.6 , 2.8 | 2.8 | 0.8 | 0.5 |
| Obstetric | Lateral | 0.15, 0.18 | 0.2 | 0.25 | 1.0 |
| Abdomen | Postero-Anterior | 0.12, 0.17 | 0.15 | 0.15 | 0.2 |
| *From Adrian Committee survey measurements nation-wide* | | | | | |
| | | | e | f | e |
| Pelvimetry[d] | | | 0.89 | 1.12 | 0.75 |
| Obstetric abdomen | | | 0.72 | 0.50 | 0.37 |

[a]Dose for vertex presentation with fetal gonads respectively 5 or 8 cm below the surface of phantoms (plaster casts at term of abdomen of one small and one large pregnant woman) (Table I, Bewley *et al.*, 1957). [b]Average dose allowing for relative frequency of vertex and breech presentations for inlet view with 'effective depth' of gonads 7 cm: the averaging process is necessarily extremely rough (Table I and text, Bewley *et al.*, 1957). [c]Table II, Bewley *et al.* (1957). [d]Table XVI classifies views in pelvimetry giving associated doses measured in 1958. [e]Table I, Ministry of Health (1960). [f]Table II, Ministry of Health (1966) and Table XVII.

pelvimetric projections but only from 0.8 to 1.0 for lateral and PA obstetric abdomen views (Bewley *et al.*, 1957). The ratio of the Adrian survey mean values was 0.8 for pelvimetry and 1.4 for obstetric abdomen (Table XVIII). The ratio of gonad to whole body dose in the fetus for different pelvimetric projections is directly correlated with magnitude of fetal gonad dose. The highest ratio 3.5 corresponds to the highest fetal gonad dose 2.8–4.6 R (for projection 4, Table XVI) and the lowest ratio 0.2 with the lowest fetal gonad dose 0.01–0.02 R (for projection 3, Table XVI). The mix of projections can be different in individual pregnant women. So 'dose from pelvimetry' is an uncertain basis for estimating risk of childhood cancer.

### Differential radiosensitivity according to stage of development *in utero*

In the years 1944–78, 90–95% of all X-raying in pregnancy was in the third trimester (Table XIV), 92, 89, 95 and 95% for birth years 1939–49, 1950–9, 1960–9 and 1970–81 (Gilman *et al.*, 1989*b*).

As would be anticipated, X-raying involving conceptus and embryo was mainly for non-obstetric reasons (Table XIV). For examinations in the first 0–7 weeks post-conception (2–9 weeks after the last menstrual period) the case/control ratio for X-raying was 4.4 for non-obstetric X-rays. This could reflect either an increased intrinsic sensitivity to X-rays in early pregnancy or a higher dose in non-obstetric examinations.

Stewart and Kneale (1970*b*) noted that 'the "extra" cancer risk for children X-rayed within 3 months of conception was more a dose effect than a susceptibility effect'. Over 50% of first trimester examinations of cancer cases and controls involved more than four films compared with 20% and 6% respectively for second and third trimester examinations (*loc. cit.*). However, soon afterwards, Stewart (1971), after writing that first trimester exposures are more dangerous than later exposures, continued 'an immature foetus is more vulnerable to the tumour induction effects of radiation than a mature foetus'. This does not follow unless the 'extra' cancer risk is too large to be explained by the 'extra' dose associated with the 'extra' film number per examination plus the additional dose from fluoroscopy when contrast media are used (but not included in the OSCC assessments of dose based on dose per film and film number).

Dose from fluoroscopy (unlike radiography) cannot be standardised. Dose per minute in tissue depends on emission rate from the X-ray tube and the duration of a fluoroscopy varies characteristically between individual radiologists (Osborn, 1963). Normally no information is recorded at the time of a fluoroscopy that would allow an estimate of radiation dose in the subject on that particular occasion. The care taken by the radiologist in coning the field of view and avoiding exposure of the uterus is possibly the crucial factor determining intrauterine dose.

First trimester examinations used four to five films per examination (Table XIX), 2–3 times more than in third trimester obstetric X-rays (Table XI). About one in four of non-obstetric X-rays in early pregnancy involved fluoroscopy (Table XIV), dose from fluoroscopy is likely to be higher than for any number of films, and OSCC assessments of dose have not included any dose from this source. Non-obstetric X-ray examinations were more frequent in future cancer cases than controls, 10.5% and 1.9%, only in the earliest years 1939–49, when doses were presumably relatively high. In 1950–81 frequencies were similar, 4.9 and 4.1% (records of reason for X-ray in Table 4, Gilman *et al.*, 1989*b*). These factors taken together show that fetal dose in OSCC was markedly higher for non-obstetric than obstetric X-rays, i.e. markedly higher for X-raying in early pregnancy than in late pregnancy.

Data for X-raying at different times within the first trimester, when nearly all X-rays were for non-obstetric pur-

**Table XIX** Number of future cancer cases/future controls with dated X-rays in early pregnancy (Oxford Survey of Childhood Cancer data)

| | | *Month of pregnancy* | | | *First trimester* | |
|---|---|---|---|---|---|---|
| *Birth years* | *Death years* | *First* | *Second* | *Third* | *Total* | *Films per examination* |
| [a]not stated | [b]1953–67 | 11/0 | 11/0 | 16/4 | 38/4 | 4.78 |
| [c]1944–78 | 1953–79 | 15/4 | 14/4 | 22/7 | 51/15[d] | 4.63 |
| 'by difference' | | 4/4 | 3/4 | 6/3 | 13/11 | |

[a]In the first trimester zero rate of dated X-rayings is reported for the controls of birth years 1939–49 and 8, 4 and 2 controls were X-rayed in 1950–9, 1960–9 and 1970–81 respectively (Table 3, Gilman *et al.*, 1989*b*), suggesting that birth years for the first row of Table XIX did not extend past 1959. [b]from Table XII in Bithell & Stewart (1975). [c]from text and Table 5 in Gilman *et al.* (1988) assuming that weeks 0–5, 6–9 and 10–13 correspond with the first, second and third month of pregnancy in Table XII in Bithell & Stewart (1975) and excluding the 4 cancer cases in the top row and 1 control in the second row of Table 5 in Gilman *et al.* (1988), cf. footnotes [b,c] in Table XIV. The total number of controls with dated X-rays in the first trimester is then 15. [d]Table 3 (Gilman *et al.*, 1989*b*) stated that 1.2% of 1133 = 14 controls were X-rayed in the first trimester. The rate should have been 1.32% of 1133 = 15 controls (Knox *et al.*, 1989, personal communication).

poses, are given in Table XIV. Risk (case/control ratio) was not higher in the first few weeks of intrauterine development. Reports in 1975 and 1988 (footnotes b and c, Table XIX) gave different case/control ratios for X-raying in the first trimester, 9.5 and 3.4, but the same film number per examination. The 1988 data are the 1975 data plus additional information. The case/control X-raying ratio for the additional information was 1.2 (cf. row labelled 'by difference', Table XIX), smaller than for birth years before 1958 (Tables VIII, IX and X), and showing that the high ratio of 9.5 was confined to early birth years. These were not given in the 1975 report but cannot have been later than 1959 (footnote a, Table XIX): most were probably earlier.

The data for X-raying in the first trimester are consistent with a substantial change in requests for radiology in the late 1950s or late 1940s. After 1949 X-rays for non-obstetric reasons were no longer 5 times more frequent in future cases than future controls (Gilman *et al.*, 1989*b*). Alternatively some of the difference between the 1975 and 1988 reports may be related in some way to the high proportion of pre-1960 deaths with incomplete X-ray records (Kneale & Stewart, 1976*b*).

Some women had several X-ray investigations during the same pregnancy. The data relating to the first X-raying were used when analysing the dose response and the timing of X-ray (Bithell & Stewart, 1975). This procedure assumes that the earlier stages of pregnancy *in utero* are the most sensitive to cancer induction, for which, as has been seen, there is no dependable evidence. A valid comparison (not yet made) would be between subjects having only a single X-ray examination in pregnancy, some early and some late.

The concept underlying the so-called ten day rule, the need to minimise X-raying in the first two post-conception weeks, was introduced by the International Commission on Radiological Protection in 1959 in the context of occupational exposure. The 'rule' was formalised for medical radiography in Britain by a DHSS recommendation in 1972 (now superseded). It was not based on fear of cancer but on a mistaken belief that the human conceptus is sensitive to induction of malformations by irradiation (Mole, 1987*a*).

Emphasising a supposed sensitivity to radiation carcinogenesis at the earliest stages of human development *in utero* has distracted attention from the fact that 95% of obstetric X-rays are in the third trimester. Reducing X-raying in the third trimester would reduce radiation-induced childhood cancer. If third trimester diagnostic X-rays have contributed to the progressively decreasing perinatal mortality over recent decades, the desirable degree of reduction in X-raying depends on balancing risk and benefit to children yet to be born.

The embryo is the stage of development during which organ primordia are laid down. Most childhood cancer (apart from leukaemia) can be classified by organ of origin. Do all classifiable cancers originate after the corresponding organ primordium has formed? A characteristic burst of cell division occurs in all mammalian embryos soon after the primitive streak becomes evident, in humans in the third week post-conception. Any cell in an early embryo already transformed by carcinogenic action would participate in this outburst of division, leading to death within a few days and loss of pregnancy rather than from cancer diagnosed in childhood. Judged by cancer deaths in childhood, radiography in the first few weeks post-conception should be less, rather than more, risky than in later pregnancy.

### Radiation dose per X-ray film an inadequate basis for risk estimation

All older and newer assessments of risk factors for carcinogenesis by fetal irradiation use, as a surrogate for fetal tissue dose, the product of film number per X-ray examination and a common value of dose per X-ray film for obstetric X-ray examinations of all kinds at a given date. This approach seems no longer justifiable.

The earliest risk assessment, by Stewart and Kneale (1970*a*), used dose estimates per film decreasing systematically from 460 to 200 mrad over the years 1943–65 but the basis for these values and for the change over time has never been published. Gonad and whole body dose were not distinguished. In 1958 fetal gonad dose per film was 600 and 520 mR for obstetric abdomen and pelvimetry respectively (Table I and Appendix Table 11, Ministry of Health, 1960). These values are double the 250 mR per film for 'mean fetal dose' in 1955–59 used by Stewart and Kneale (1970*a*). That lower value had been provided by Dr G.M. Ardran after consideration of radiological practice and the literature. 'The accuracy of the Ardran estimates . . . is an unknown quantity' (Stewart & Kneale, 1973).

Values for fetal dose in obstetric radiography in Britain over the 23 years 1943–65 were given by UNSCEAR (1972), mean dose per film decreasing from 1,800 to 200 mrad. All UNSCEAR values were said to be derived from the British literature, the latest citation dated 1957 but dose given up to 1965. All cited references were to studies in teaching hospitals: doses there cannot be accepted as average values for all Britain. Adrian Committee Reports (1960, 1966) were not listed. These unjustified UNSCEAR values, and the mistaken assumption that cancer risk is directly dependent on film number per X-ray examination, were the basis for risk factors derived by UNSCEAR (1972) and 16–17 years later by Bithell and Stiller (1988) and Muirhead and Kneale (1989).

Table XVII gives unpublished information from the Adrian survey on fetal whole body dose per X-ray film. Dose per film is far from constant. When the Adrian survey measurements were made in 1958 the fetal dose per film for pelvimetry using a single film was more than twice as high as for pelvimetry using multiple films. It was more nearly independent of number of films in obstetric abdomen examinations (Table XVII).

Primary data on film number per X-ray were always less adequate than for other OSCC observations. Some numbers were recorded, some were estimates many years in retrospect about how many films were thought to have been used. Information was missing for an unstated proportion of subjects. A larger mean film number for X-rayed cancer cases than for X-rayed controls was found only in early birth years of the OSCC (Figure 2; Gilman *et al.*, 1989*b*). Updated OSCC analysis no longer shows any association between cancer risk and number of films per X-ray obtained from medical records (Table XII; Gilman *et al.*, 1988).

It is wrong in principle to expect a common value of fetal dose per X-ray film, independent of the purpose of an obstetric X-ray examination and of the geometric relationships of X-ray tube focus, X-ray beam, the body of the fetus, the maternal abdomen and the X-ray film. The range of mean fetal gonad dose for differing projections in pelvimetry was 16-fold (Table XVI). Dose reduction by ceasing to use Thoms' view, when fetal dose in routine pelvimetry exceeds 2,000 mR for a single film, was considerably greater than by reducing number of X-ray films per pelvimetry by one. In Britain Thom's view had been virtually abandoned by 1958, the year when mean fetal gonad dose in pelvimetry was 885 mR, when only 15 of 110 determinations exceeded 2,000 mR (Figure 5F, Appendix I, Ministry of Health, 1960) and only 10 of the 15 were Thoms' inlet view (Table XVII, footnote d).

Modern statistical developments may allow the quantitative importance of individual carcinogenic factors to be distinguished by stratified analyses and were applied in recent derivations of risk factors for obstetric radiography (Bithell & Stiller, 1988; Muirhead & Kneale, 1989). But analyses based on the assumption that dose per film is a constant at a given date and that its product with number of films per examination is an adequate surrogate for fetal tissue dose cannot be trustworthy, however sophisticated the analyses may be in other respects.

**Evidence that prenatal X-ray exposure is a cause of childhood cancer**

In the 1950s and 1960s the dogma that genetic damage depended linearly on gonad dose and without a dose threshold was never criticised. But until fairly recently the application of the corresponding hypothesis to cancer induction by radiation was strongly resisted. Indeed Stewart and Kneale's initial finding (1970*a*) of a quantitative relationship between rate of excess cancer and number of films per X-ray examination in OSCC data seemed at the time to be the first direct evidence in man that linearity without threshold for radiation carcinogenesis might have some plausibility.

*Observations on twins*

A cogent and independent line of evidence, based on OSCC observations but independent of film number and radiation dose, shows that prenatal exposure to diagnostic X-ray examinations can cause childhood cancer. Excess rates of childhood leukaemia and cancer in the X-rayed were virtually the same in singleton births and in twins although 10% of singletons and 50–60% of twins were irradiated (Mole, 1974). Independently of the gross difference in proportion of subjects X-rayed, the same number of excess cancers was found when the same number of fetuses, singletons or twins, were exposed (presumably) to the same dose. This is as predicted if X-raying is causal but not if mothers selected for X-raying were already destined to have children with an above average cancer rate.

Past findings in twins cannot be compared with updated OSCC information limited to singleton births (Knox *et al.*, 1987; Gilman *et al.*, 1988). Data for twins set out as for singletons in Tables I–VII would be useful. In NBT data 50–60% of twins were X-rayed *in utero* in 1958 (Stewart, 1973) and even more, 73%, in 1970 (Dr J. Golding, personal communication, 1989).

Confirmatory evidence from USA showed an excess of childhood cancer in irradiated twins (relative risk (RR) 2.4 with 95% CI 1.0–5.9, Harvey *et al.*, 1985). The corresponding data on singleton births in an extended USA survey of childhood cancer and X-raying in pregnancy showed a significant association between leukaemia frequency and intrauterine X-ray exposure (RR 1.52 with 95% CI 1.18–1.95) but not for solid tumours (RR 1.3, lower 95% CI 0.95). (Monson & MacMahon, 1984; MacMahon, 1985). The excess risk (RR – 1.0) is much higher in twins than singletons, as predicted by the causal hypothesis, but the CI of each RR are much too wide for definite conclusions: the population sample in USA was much smaller than in Britain. A factor affecting comparisons is that in Britain rates for leukaemia and solid cancers in the unirradiated were each smaller in twins than in singletons (Mole, 1974).

MacMahon was reluctant to accept that prenatal X-raying did cause cancer. Being (or being suspected of being) 'a twin no doubt accounts for the substantially higher frequency of X-ray exposures in twin pregnancies. But the fact of a twin pregnancy did not exclude all other indications for radiography; one of these may have been the mysterious third factor, and it could operate in both single and twin pregnancies' (MacMahon, 1985). This is saying merely that causation by X-rays need not be the only factor in an association of prenatal X-rays and extra childhood cancer. This cannot be denied: it is clear that proof of causation cannot of itself disprove the existence of some other factor and vice versa (Mole, 1974). Doll (1981) and MacMahon himself (1985) stressed that this theoretical 'third' factor remained elusive in spite of intensive attempts to unearth it.

*Correlated change in excess cancer and X-raying rate*

A reduction in fetal radiation dose from obstetric radiography, beginning suddenly in 1957/8, was associated with a corresponding and significant reduction in odds ratio for childhood cancer mortality in children born during the next 8–12 years (Table XIII). The motive for decreasing fetal radiation dose was primarily to reduce population gonad dose and, therefore, to reduce hereditary damage. A reduction in childhood cancer associated with reduction in dose from medical radiology in the face of disbelief that low doses of radiation could cause cancer may be in some sense a serendipitous event but that only reinforces the strong inference that fetal irradiation by medical radiography is truly carcinogenic.

If diagnostic radiography does cause cancer, the increase in rate of X-raying of early 1970 births (Figure 1) would tend to increase childhood cancer. Cancer deaths at 0–5 years old did increase in 1970–6 births as compared with 1958–69 births (Table VIII) but not significantly. Most OSCC cancer data for birth years 1970 onwards are not yet published (Tables VIII, IX and X). Conclusive evidence that diagnostic X-rays do cause cancer would be a marked decrease in childhood cancer in those born most recently and whose antenatal care involved ultrasound rather than X-rays.

**Carcinogenic risk of irradiation *in utero***

When observations are collected over several decades pooling the data may conceal discontinuous step-like changes. Such changes occurred before 1950 in the case–control ratio of film number per examination (Figure 2) and in requests for radiology in pregnant women for non-obstetric reasons, and in the late 1950s in mean number of films per X-ray examination (Table XI; Adrian survey) and in the case–control ratio of X-raying rate (Figure 1). In the late 1950s the abrupt changes were the result of pressure to reduce fetal gonad dose for fear of genetic hazards. I was wrong to infer (Mole, 1989) that the dating of the change indicated a response to the first OSCC publications showing an association between excess childhood cancer and diagnostic X-raying in pregnancy.

When substantial changes occur in diagnostic radiography in a discontinuous manner and radiation dose is to be correlated with cancer mortality (or incidence), it is essential to derive the data to be compared from the same calendar period. In fact the only nationwide measurements of dose in obstetric radiography in Britain with which to compare OSCC cancer data are those made in 1958 in the course of the Adrian survey. The relevant dose is mean dose in the fetal body. This, not gonad dose, is the basis for carcinogenesis by prenatal irradiation.

*A risk co-efficient for carcinogenesis by diagnostic radiography of the fetus*

The OSCC category 'all malignant tumours' included CNS tumours (Bithell & Stewart, 1975). National data on deaths from malignant neoplasms for 1952–60 births (Draper *et al.*, 1982) excluded all other CNS, intracranial and intraspinal tumours because these are sometimes without histological confirmation. The Childhood Cancer Research Group, University of Oxford, has kindly provided data for birth years 1958–72. Deaths from malignant neoplasms alone and combined with deaths from all other tumours at ages 0–14 years are given in Table XX with corresponding population rates.

Death rates at ages 0–14 for birth years 1958, 1959 and 1960 were the same (Table XX). In 1961 the childhood cancer death rate decreased by 8–9% and continued to decrease progressively during the next decade, presumably as a result of improved therapy. Mean death rate at ages 0–14 for the three birth years 1958, 1959 and 1960 was 112.8 per 100,000 for malignant neoplasms plus all other CNS, etc., tumours. When increased by 15/14 this gives a lethal tumour rate at ages 0–15 years = 12.1 per 10,000 per year.

OR after X-raying *in utero* in Britain in the four birth years 1958–61 was 1.27, 1.36 and 1.02 for cancer deaths at ages 0–5, 6–9 and 10–15 respectively (Tables VIII, IX and X). OR = 1.23 for all ages 0–15 years, with 95% CI 1.04–1.48. Thus the excess lethal tumour rate from X-raying

**Table XX** Cancer death rates in Great Britain by year of birth 1958–72 at ages 0–14 years (data from C.A. Stiller, personal communication, 1989)

| | | *Number of deaths aged 0–14* | | *Rate per 10,000* | | | |
|---|---|---|---|---|---|---|---|
| | | | | *Malignant neoplasms* | | *Malignant plus all other CNS neoplasms*[a] | |
| *Birth year* | *Number of births* | *Malignant neoplasms* | *Malignant plus all other CNS neoplasms*[a] | *per year* | *4-year average* | *per year* | *4-year average* |
| 1958 | 840,196 | 921 | 977 | 10.96[b] | | 11.63 | |
| 1959 | 847,752 | 856 | 929 | 10.10[b] | 10.32 | 10.96 | 11.02 |
| 1960 | 886,297 | 948 | 998 | 10.70[b] | | 11.26 | |
| 1961 | 912,450 | 868 | 934 | 9.51 | | 10.24 | |
| 1962 | 943,070 | 879 | 937 | 9.32 | | 9.94 | |
| 1963 | 956,746 | 903 | 972 | 9.44 | 9.15 | 10.16 | 9.85 |
| 1964 | 980,327 | 890 | 947 | 9.08 | | 9.86 | |
| 1965 | 963,385 | 842 | 910 | 8.74 | | 9.45 | |
| 1966 | 946,359 | 836 | 899 | 8.83 | | 9.50 | |
| 1967 | 928,385 | 843 | 907 | 9.08 | 8.50 | 9.77 | 9.14 |
| 1968 | 914,058 | 739 | 794 | 8.08 | | 8.69 | |
| 1969 | 887,828 | 710 | 763 | 8.00 | | 8.59 | |
| 1970 | 871,821 | 689 | 756 | 7.90 | | 8.67 | |
| 1971 | 869,883 | 674 | 725 | 7.75 | (7.72) | 8.33 | (8.28) |
| 1972 | 803,990 | 604 | 625 | 7.51 | | 7.77 | |

[a]includes additonally all deaths from benign and unspecified CNS/intracranial/intraspinal tumours. For tumours in these sites without histology the distinction between malignant and non-malignant is somewhat arbitrary. [b]The mean of the tabulated values for 1958–60 is 10.6. The rates in Draper *et al.* (1982) are 10.83, 10.05 and 10.55 (mean 10.5) respectively. The small differences between the published rates and those tabulated here originate in reclassification of some neoplasms.

*in utero* was $0.23 \times 12.1 \times 10^{-4} = 2.8 \times 10^{-4}$ with 95% CI $0.48–5.8 \times 10^{-4}$, using the 3-year mean national death rate as the base line.

Mean fetal whole body dose in 1958 was 0.5 rad for obstetric abdomen and 1.12 rad for pelvimetry (Table XVIII). There were 0.8 examinations per 1,000 persons for the former, 0.19 for the latter (Table AII, Ministry of Health, 1966), giving a weighted mean 0.61 rad for whole body dose in irradiated fetuses from all obstetric radiography. This value for fetal dose can be taken to apply over the four birth years 1958–61, given that changes from 1958 to 1964 and subsequently were small, as discussed earlier. An excess cancer death rate $2.8 \times 10^{-4}$ caused by 0.61 cGy gives a risk coefficient $4.6 \times 10^{-4}$ per cGy with 95% CI $0.8–9.5 \times 10^{-4}$ per cGy. It applies directly to X-raying in the third trimester (cf. Table XIV) and to deaths at ages 0–15.

This seems to be the only value for risk of cancer mortality after irradiation *in utero* based on independent determinations of dose and of risk in nationwide samples of the same population of subjects. It is not based on extrapolation or an unreliable dose-response. It applies equally to cancer incidence and cancer mortality at ages 0–15 years because incidence and mortality were the same.

The mean cancer rate for the four birth years 1958–61 = 11.02 deaths per 100,000 (Table XX). The risk co-efficient derived as before has the value $4.5 \times 10^{-4}$ per cGy, virtually equal that derived above using a three birth year mean. Whether the slight reduction in lethal tumour rate for the birth year 1961 as compared with 1958–60 is attributable to therapy, or is a chance finding, it has virtually no influence on the value of a risk co-efficient for induction of lethal tumours by radiography *in utero*.

*Japanese bomb survivors irradiated in utero*

Two cancers (neither leukaemia) occurred at ages 0–15 years: one subject died with liver cancer and one continued to survive having had Wilm's tumour. The apparently low rate of childhood cancer after exposure to bomb radiation has often been regarded as conflicting with the higher rate found after prenatal medical radiology. Statistical and radiobiological considerations show that such an inference would be a mistake (Mole, 1974; UNSCEAR, 1977). It continues to be made (e.g. in UNSCEAR, 1988).

The upper limit of the two-tailed 95% CI for risk based on the two cancer cases observed at ages 0–14 years is $2.79 \times 10^{-4}$ per population-cGy DS86 dose (Yoshimoto *et al.*, 1988) and for one cancer death is $2.2 \times 10^{-4}$ per cGy (the 95% upper CI for two and one are 7.2 and 5.6 respectively). Both values are well within the 95% CI ($0.9–9.5 \times 10^{-4}$ per cGy) for the risk coefficient derived here for diagnostic X-rays and applicable to both cancer mortality and incidence at 0–15 years of age.

Much of the total population dose in bomb survivors irradiated *in utero* came from the highest dose group (Yoshimoto *et al.*, 1988). Its exposure was at levels that make obligatory an allowance for inactivation of transformed cells by the same dose that was responsible for the transformation (Mole 1974, 1984). If standard radiosensitivity of cells is assumed ($b$ in $e^{-bD} = 0.01$ cGy$^{-1}$), the risk coefficients for both childhood cancer mortality and incidence in bomb survivors irradiated *in utero* would be larger by 2 times (or more) (judging by the distribution of T65D doses, Mole, 1974). This would make the apparent difference between bomb radiation and medical radiology even smaller. If fetal cells are thought to be more sensitive to inactivation by radiation than cells in the adult, the corrected value for risk in bomb survivors would be further increased.

No case of childhood leukaemia was seen in bomb survivors exposed *in utero*. The 95% upper Poisson limit for zero is 3.7, 2/3 of the value 5.6 for one case, and the excess of leukaemia after prenatal X-raying is about half that for all childhood cancers (Bithell & Stewart, 1975). The same arguments as for all cancers show that an absence of childhood leukaemia in bomb survivors exposed *in utero* is also not a genuine discrepancy.

I am very indebted to those who initiated and maintained the Oxford Survey of Childhood Cancer, to Dr Alice Stewart in the first place, and to Professor George Knox, Dr George Kneale, and Miss Estelle Gilman, her current colleagues. The unpublished information they have given me is referred to in the text and tables as a personal communication from Knox *et al.* (1989). They are not responsible in any way for the use I have made of the data. I consulted many people, those who provided unpublished information cited as personal communications and others too many to thank by name. Dr Sidney Osborn's thesis (1960) was an essential source of contemporary information. Mr David Papworth, Medical Research Council Radiobiology Unit, Chilton, gave me statistical help. Dr G.J. Draper, Director, and Dr C.A. Stiller, Childhood Cancer Research Group, University of Oxford, kindly provided unpublished tables of national records of deaths from childhood cancer by year during 1953–72.

### References

BEWLEY, D.K., LAWS, J.W. & MYDDLETON, C.J. (1957). Maternal and foetal radiation dosage during obstetric radiographic examinations. *Br. J. Radiol.*, **30**, 286.

BITHELL, J.F. & STEWART, A.M. (1975). Pre-natal irradiation and childhood malignancy: a review of British data from the Oxford survey. *Br. J. Cancer*, **31**, 271.

BITHELL, J.F. & STILLER, C.A. (1988). A new calculation of the carcinogenic risk of obstetric X-raying. *Stat. Med.*, **7**, 857.

BLAIR HARTLEY, J. (1956). Radiation hazards. *Lancet*, **ii**, 46.

BROWNE, F.J. (1951). *Antenatal and Postnatal Care*, 7th edition, p. 44. J. & A. Churchill: London.

BUTLER, N.R. & BONHAM, D.G. (1963). *Perinatal Mortality*. E. & S. Livingstone: Edinburgh.

CARMICHAEL, J.H.E. & BERRY, R.J. (1976). Diagnostic X-rays in late pregnancy and in the neonate. *Lancet*, **i**, 351.

CHAMBERLAIN, G., PHILLIP, E., HOWLETT, B. & MASTERS, K. (1978). *British Births, 1970, vol. 2, Obstetric care*, p. 24. Heinemann Medical: London.

CLARK, K.C. (1956). *Positioning in Radiography*, 7th edition. Heinemann Medical: London.

CLARK, K.C. (1964). *Positioning in Radiography*, 8th edition. Heinemann Medical: London.

CLAYTON, C.G., FARMER, F.T. & WARRICK, C.K. (1957). Radiation doses to the foetal and maternal gonads in obstetric radiography during late pregnancy. *Br. J. Radiol.*, **30**, 291.

DOLL, R. (1981). Radiation hazards: 25 years of collaborative research. *Br. J. Radiol.*, **54**, 179.

DRAPER, G.J., BIRCH, J.M., BITHELL, J.F., KINNIER-WILSON, L.M. & LECK, I. (1982). *Childhood Cancer in Britain: Incidence, Survival and Mortality*. OPCS: London.

GILMAN, E.A., KINNIER WILSON, M., KNEALE, G.W. & WATERHOUSE, J.A.H. (1989*a*). Childhood cancers and their association with pregnancy drugs and illnesses. *Paediatr. Perinatal Epidemiol.*, **3**, 66.

GILMAN, E.A., KNEALE, G.W., KNOX, E.G. & STEWART, A.M. (1988). Pregnancy X-rays and childhood cancers: effects of exposure age and radiation dose. *J. Radiol. Prot.*, **8**, 3.

GILMAN, E.A., STEWART, A.M., KNOX, E.G. & KNEALE, G.W. (1989*b*). Trends in obstetric radiography. *J. Radiol. Prot.*, **9**, 93.

HARVEY, E.B., BOICE, J.D., HONEYMAN, M. & FLANNERY, J.T. (1985). Prenatal X-ray exposure and childhood cancer in twins. *N. Engl. J. Med.*, **312**, 541.

HEWITT, D, SANDERS, B. & STEWART, A. (1966). Oxford survey of childhood cancers: Progress Report IV - Reliability of data reported by case and control mothers. *Bull. Min. Health*, **25**, 80.

JOINT COMMITTEE OF THE ROYAL COLLEGE OF OBSTETRICIANS AND GYNAECOLOGISTS AND THE POPULATION INVESTIGATION COMMITTEE (1948). *Maternity in Great Britain*. Oxford University Press: London.

KENDALL, G.M., DARBY, S.C., HARRIES, S.V. & RAE, S. (1980). *A Frequency Survey of Radiological Examinations Carried out in National Health Service Hospitals in Great Britain in 1977 for Diagnostic Purposes*. National Radiological Protection Board Report NRPB R-104. HMSO: London.

KENDALL, G.M., WALL, B.F. & DARBY, S.C. (1989). X-ray exposures of the foetus. *J. Radiol. Prot.*, **9**, 285.

KNEALE, G.W. & STEWART, A.M. (1976*a*). Mantel–Haenszel analysis of Oxford data. I. Independent effects of several birth factors including fetal irradiation. *J. Natl Cancer Inst.*, **56**, 879.

KNEALE, G.W. & STEWART, A.M. (1976*b*). Mantel–Haenszel analysis of Oxford data. II. Independent effects of fetal irradiation subfactors. *J. Natl Cancer Inst.*, **56**, 1009.

KNEALE, G.W. & STEWART, A.M. (1980). Pre-conception X-rays and childhood cancers. *Br. J. Cancer*, **41**, 222.

KNOX, E.G., STEWART, A.M., KNEALE, G.W. & GILMAN, E.A. (1987). Prenatal irradiation and childhood cancer. *J. Radiol. Prot.*, **7**, 177.

MACMAHON, B. (1985). Prenatal X-ray exposure and twins. *N. Engl. J. Med.*, **312**, 576.

MARTIN, J.H. & WILLIAMS, E.R. (1946). A note on the amount of radiation incident in the depths of the pelvis during radiological pelvimetry. *Br. J. Radiol.*, **19**, 297.

MATTHEWS, J.C. & MILLER, H. (1969). Radiation hazards from diagnostic radiology. A repeat survey over a small area. *Br. J. Radiol.*, **42**, 814.

MEDICAL RESEARCH COUNCIL (1956). *The Hazards to Man of Nuclear and Allied Radiations*. HMSO: London.

MINISTRY OF HEALTH (1960). *Radiological Hazards to Patients*. Second Report. HMSO: London.

MINISTRY OF HEALTH (1966). *Radiological Hazards to Patients*. Final Report. HMSO: London.

MOIR, J.C. (1951). The uses and value of radiology in obstetrics. In *Antenatal and Postnatal Care*, 7th edition, Browne, F.J. (ed.) p. 642. J. & A. Churchill: London.

MOIR, J.C. (1955). The uses and value of radiology in obstetrics. In *Antenatal and Postnatal Care*, 8th edition, Browne, F.J. & Browne, J.C.M. (eds) p. 614. J. & A. Churchill: London.

MOIR, J.C. (1956). Radiation hazards. *Lancet*, **ii**, 99.

MOIR, J.C. (1960). The uses and value of radiology in obstetrics. In *Antenatal and Postnatal Care*, 9th edition, Browne, F.J. & Browne, J.C.M. (eds) p. 389. J. & A. Churchill: London.

MOLE, R.H. (1974). Antenatal irradiation and childhood cancer: causation or coincidence? *Br. J. Cancer*, **30**, 199.

MOLE, R.H. (1984). Dose–response relationships. In *Radiation Carcinogenesis: Epidemiology and Biologic Significance*, Boice, J.D. & Fraumeni, J.F. (eds) p. 403. Raven Press: New York.

MOLE, R.H. (1987*a*). The so-called 10-day rule. *Lancet*, **ii**, 1138.

MOLE, R.H. (1987*b*). Irradiation of the embryo and fetus. *Br. J. Radiol.*, **60**, 17.

MOLE, R.H. (1989). Carcinogenesis following medical uses of ionizing radiation. In *Low Dose Radiation: Bases of Risk Assessment*, Baverstock, K.F. & Stather, J.W. (eds) p. 100. Taylor & Francis: London.

MONSON, R.R. & MACMAHON, B. (1984). Prenatal X-ray exposure and cancer in children. In *Radiation Carcinogenesis: Epidemiology and Biologic Significance*, Boice, J.D. & Fraumeni, J.F. (eds) p. 97. Raven Press: New York.

MORRIS, J.A. & GARDNER, M.J. (1988). Calculating confidence intervals for relative risks (odds ratios) and standardised ratios and rates. *Br. Med. J.*, **i**, 1313.

MUIRHEAD, C.R. & KNEALE, G.W. (1989). Prenatal irradiation and childhood cancer. *J. Radiol. Prot.*, **9**, 209.

MÜLLER, H.J. (1954). The manner of dependence of the 'permissible dose' of radiation on the amount of genetic damage. *Acta Radiol.*, **41**, 5.

OSBORN, S.B. (1951). Radiation doses in radiographic pelvimetry. *Br. J. Radiol.*, **24**, 174,

OSBORN, S.B. (1960). *A study of radiation hazards to large populations with special reference to diagnostic radiology*. PhD thesis, University of London.

OSBORN, S.B. (1961). British survey of the radiation dose to the population from medical radiology. *Transactions IXth International Congress of Radiology 1959 München*, p. 103. George Thieme Verlag: Stuttgart.

OSBORN, S.B. (1963). Variations in the radiation dose received by the patient in diagnostic radiology. *Br. J. Radiol.*, **36**, 230.

OSBORN, S.B. & SMITH, E.E. (1956). The genetically significant radiation dose from the diagnostic use of X-rays in England and Wales. *Lancet*, **i**, 949.

SANKARANARAYANAN, K. (1988). Invited review: prevalence of genetic and partially genetic disease in man and the estimation of genetic risks of exposure of ionizing radiation. *Am. J. Human Genet.*, **42**, 651.

SPIERS, F.W. (1957). Measurement of the gonadal dose in the medical use of X-rays: a preliminary report of a survey being made in the United Kingdom. *Phys. Med. Biol.*, **2**, 152.

STANFORD, R.W. (1951). Radiation doses in radiographic pelvimetry. *Br. J. Radiol.*, **24**, 226.

STEWART, A. (1971). Low dose radiation cancer in man. *Adv. Cancer Res.*, **14**, 359.

STEWART, A.M. (1973). Cancer as a cause of abortions and stillbirths: the effect of these early cancer deaths on the recognition of radiogenic leukaemias. *Br. J. Cancer*, **27**, 465.

STEWART, A. & KNEALE, G.W. (1970*a*). Radiation dose effects in relation to obstetric X-rays and childhood cancers. *Lancet*, **i**, 1185.

STEWART, A.M. & KNEALE, G.W. (1970*b*). Prenatal radiation exposure and childhood cancer. *Lancet*, **ii**, 1190.

STEWART, A. & KNEALE, G.W. (1973). Childhood cancer following obstetric radiography. *Health Phys.*, **24**, 359.

STEWART, A., WEBB, J., GILES, D. & HEWITT, D. (1956). Malignant disease in childhood and diagnostic irradiation *in utero*. *Lancet*, **ii**, 447.

STEWART, A., WEBB, J. & HEWITT, D. (1958). A survey of childhood malignancies. *Br. Med. J.*, **i**, 1496.

UNSCEAR (1972). Radiation carcinogenesis in man. Annex H para 239. In *Ionizing Radiation: Levels and Effects, vol. II: Effects*. United Nations: New York.

UNSCEAR (1977). Radiation carcinogenesis in man. Annex G para 308. In *Sources and Effects of Ionizing Radiation*. United Nations: New York.

UNSCEAR (1988). Radiation carcinogenesis in man. Annex F para 155. In *Sources, Effects and Risks of Ionizing Radiation*. United Nations: New York.

WALL, B.F., FISHER, E.S., SHRIMPTON, P.C. & RAE, S. (1980). *Current Levels of Gonad Irradiation from a Selection of Routine Diagnostic X-ray Examinations in Great Britain*. National Radiological Protection Board Report R-105. HMSO: London.

WILLIAMS, E.R. (1950). Techniques for the study of foetal-pelvic proportions – pelvimetry – cephalometry – pelvioradiography. In *A Textbook of X-ray Diagnosis by British Authors, vol. 3*, 2nd edition, Shanks, S.C. & Kerley, P. (eds) p. 598. H.K. Lewis: London.

YOSHIMOTO, Y., KATO, H. & SCHULL, W.J. (1988). Risk of cancer among children exposed *in utero* to A-bomb radiations, 1950–84. *Lancet*, **ii**, 665.